# Comparative Morphology and Mechanics of Aquatic and Terrestrial Feeding Salamanders

**DOI:** 10.1002/jmor.70172

**Published:** 2026-09-20

**Authors:** Julia R. S. May, Laura B. Porro, Emily J. Rayfield

**Affiliations:** ^1^ Palaeobiology Research Group, School of Earth Sciences University of Bristol Bristol UK; ^2^ PalaeoHub Research Facility Hull York Medical School/University of York York North Yorkshire UK; ^3^ Centre for Integrative Anatomy, Department of Cell and Development Biology University College London London UK

**Keywords:** amphibians, anatomy, finite element analysis, jaw muscles

## Abstract

Salamanders are often used as models to understand the iconic vertebrate water–land transition as they exhibit similar morphology and, presumably, ecologies as early tetrapods. Recent studies have demonstrated previously unappreciated diversity in salamander feeding mechanisms, however, compared to mammals, and reptiles there is little data on the internal mechanical response of salamander skulls to feeding loads. In this study, we used data from microCT scans to compare skull osteology and jaw muscle architecture in an aquatic salamander (*Necturus*) and a terrestrial salamander (*Salamandra*), with resulting three‐dimensional digital models and anatomical information used to carry out lever arm and finite element analyses to predict the mechanical behaviour of the skull under bite loads. *Necturus* and *Salamandra* exhibit markedly different skull osteology in terms of overall skull shape, bones composing the skull, and dentition. Contrary to our expectations, *Necturus* exhibited both absolutely and relatively larger jaw muscles than *Salamandra* and produced higher bite forces, even when scaled to the same size. At life‐size, the skull of *Necturus* featured higher peak stresses than that of *Salamandra;* however, when scaled to the same surface area: muscle force ratio, the skull of *Necturus* featured lower peak stresses, suggesting its skull is stronger under biting loads than that of *Salamandra*. The skull of *Salmandra* did exhibit more evenly distributed stress than that of *Necturus*, suggesting skull shape in the terrestrial taxon may more closely reflect adaptation to resist biting loads. Our finite element models demonstrate that the inclusion of sutures in our models resulted in increased peak stresses; however, in terms of other performance metrics the impact of sutures on model behaviour was complex. Our results suggest that jaw muscle size, bite force and skull strength in salamanders are being driven by multiple factors, and are not solely related to differences in habitat.

## Graphical abstract

Jaw muscle size, bite force, and skull strength are driven by multiple factors, not just the difference between the two habitats. Necturus produces higher bite forces than Salamandra, but the stress distribution is less even in Necturus, suggesting Salamandra's skull shape is better adapated to resist feeding loads.

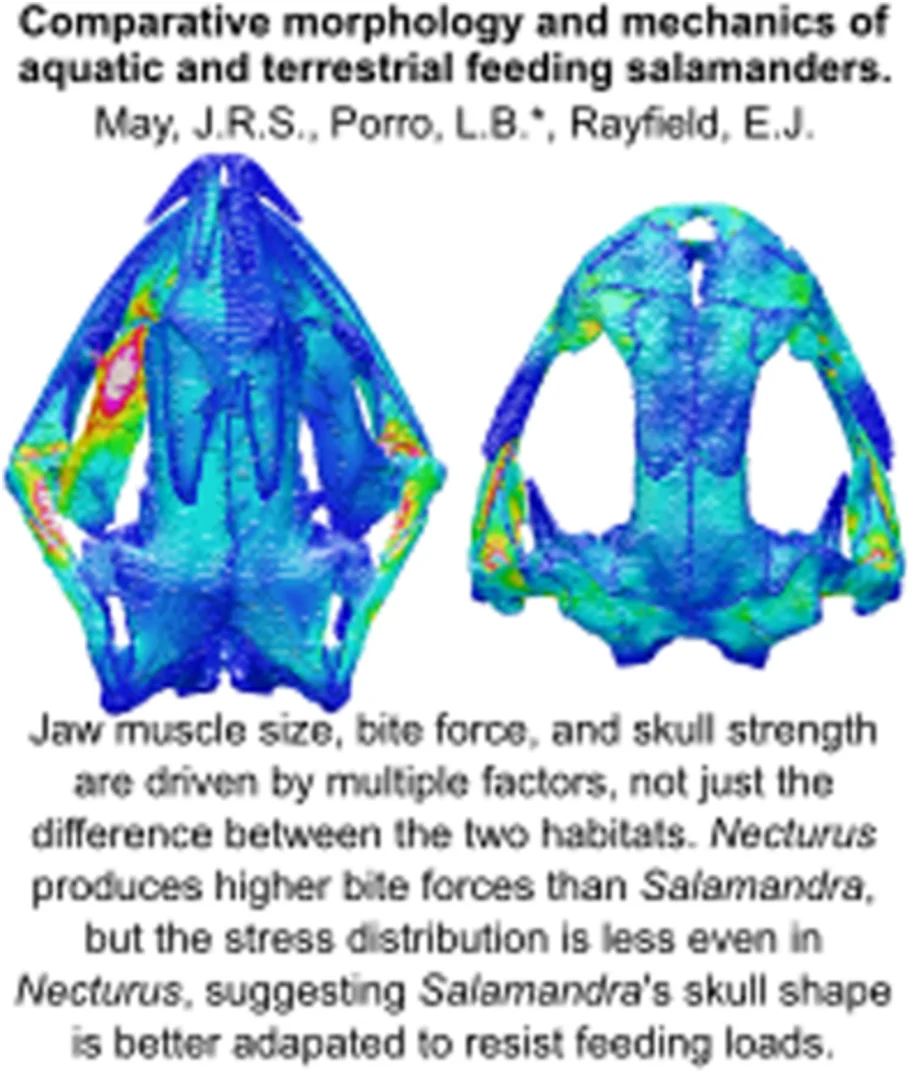

## 1 Introduction

The water–land transition was a key event in the evolution of terrestrial vertebrate life, occurring across the Late Devonian and into the Carboniferous, and requiring changes in feeding strategy, locomotion, breathing, sensory systems, and reproduction (Clack 2012; Coates et al. 2008). Feeding in particular plays a pivotal role as it strongly impacts an organism's fitness and reproductive success. Understanding the evolution of feeding adaptations that permitted tetrapods to inhabit and radiate across terrestrial environments is essential to understanding vertebrate evolution. New fossils documenting this transition have been described over the past decades (Lebedev and Coates 1995; Coates 1996; Boisvert 2005; Ahlberg and Clack 2006; Shubin et al. 2006; Pardo et al. 2017; Beznosov et al. 2019; Ahlberg and Clack 2020), with some studies inferring probable feeding mode from skull anatomy (Ahlberg and Clack 1998; Lemberg et al. 2021; Porro et al. 2023).

The greatest challenge faced by vertebrates when transitioning from aquatic to terrestrial feeding is due to the pronounced differences in the physical properties of water compared to air. Many extant aquatic organisms use suction‐feeding for prey capture and ingestion, in which rapid expansion of the oral cavity pulls prey into the mouth and digestive tract (Wainwright et al. 2015). Suction feeding is impossible on land due to the lower viscosity and density of air compared to water (Heiss et al. 2018; Van Wassenbergh 2019). Abundant evidence suggests that Devonian tetrapods (as well as many post‐Devonian forms) remained firmly tied to water while evolving traits associated with terrestrial feeding (Ahlberg and Clack 2020; Clack 2012; Schoch 2014) including changes to skull anatomy, such as: snout elongation (Clack 2012; Coates et al. 2008); reduction in the number of skull bones (Rawson et al. 2022); increased sutural complexity (Markey and Marshall 2007); changes in orbit size and orientation (MacIver et al. 2017); changes in mandibular shape and dentition (Ahlberg and Clack 1998); and reduction in the gill skeleton (Clack 2012). These morphological changes have been linked to changes in jaw‐closing musculature and jaw mechanics (Janis and Keller 2001; Olson 1961), and the first stage of a correlated shift from suction feeding to snapping and biting (Ahlberg and Clack 2020), although there has been limited biomechanical testing of these hypotheses involving fossil taxa (Anderson et al. 2013; Neenan et al. 2014; Berks et al. 2025; Ponstein et al. 2025).

In addition to examining evidence from the fossil record, a second approach to understanding how organisms overcome the challenges presented by new environments is studying modern species that span aquatic and terrestrial habitats (Ashley‐Ross et al. 2013). This includes studying terrestrial feeding in amphibious fish species (see Heiss et al. 2018; Van Wassenbergh 2019 for reviews) as well as utilising phylogenetic bracketing (Witmer and Thomason 1995) to focus on relatively closely related species. In the case of early tetrapods, these include sarcopterygian fishes (i.e., coelacanths and lungfish) and extant amphibians. Extant salamanders (Caudata) have long been used as analogues for understanding feeding in early tetrapods (Schmalhausen 1968; Carroll and Holmes 1980; Jarvik 1980; Duellman and Trueb 1986; Lauder and Reilly 1994; Kleinteich et al. 2014; Schwarz et al. 2023). This is due to: (1) broad similarities in cranial (i.e., broad, flat heads) and hyobranchial anatomy (Clack 2012; Coates et al. 2008; Jarvik 1980; Witzmann 2013); (2) many salamanders exhibiting an amphibious lifestyle analogous to that inferred for early tetrapods (Clack 2012; Schoch 2014), including forms (newts) that transition between aquatic and terrestrial environments seasonally (Heiss, Aerts, et al. 2013; Heiss and De Vylder 2016); (3) some salamanders metamorphosing from an aquatic larval stage to a terrestrial adult (e.g., *Salamandra salamandra*, the fire salamander), following the proposed evolutionary path of early tetrapods (Bonett et al. 2022; Duellman and Trueb 1986); and (4) some species in which adults remain fully aquatic due to paedomorphosis (e.g. *Necturus maculosus*, the common mudpuppy). Additionally, some salamander species feature facultative metamorphosis, with both aquatic paedomorphic and terrestrial metamorphic adult forms that exhibit different morphologies (Heiss and Grell 2019; Schwarz, Konow, Porro, et al. 2020). For all of these reasons, salamanders may provide insights into adaptations in morphology and behaviour required for the water–land transition (Heiss et al. 2018; Schwarz et al. 2023).

Diversity in the morphology of the salamander feeding apparatus facilitates or necessitates diversity in feeding mechanisms, which have been studied using a breadth of experimental techniques and have been the focus of several recent reviews (Heiss et al. 2018; Schwarz et al. 2023, 2026). Many salamanders capture and ingest prey using suction powered by the hyobranchial or hyolingual systems (Lauder and Shaffer 1985; Lauder and Reilly 1988; Reilly and Lauder 1989a; Reilly 1995; Kleinteich et al. 2014; Heiss and Lemell 2023; Toussaint‐Lardé et al. 2025) although the Chinese and Japanese giant salamanders use an alternative mechanism that relies on rapid opening of the plate‐like jaws to generate suction, perhaps analogous to mechanisms used by early tetrapods (Heiss, Natchev, et al. 2013; Matsumoto et al. 2024). Other species in both aquatic and terrestrial habitats seize prey in their jaws and may engage in head‐shaking (Lukanov et al. 2016) or twist feeding to subdue prey (Cagle 1948), or—in the case of certain plethodontids—biting prey into pieces (Dauth 1983). Terrestrial‐feeding newts as well as most metamorphic salamanders use hyolingual prehension, in which the tongue is used to grasp prey (Reilly and Lauger 1989b; Heiss and De Vylder 2016; Wake and Deban 2000), with some plethodontids evolving ballistic tongue feeding (Deban et al. 1997). In terms of food processing, Schwarz et al. (2023, 2026) identified three developmental strategies associated with different processing behaviours. Early larval forms as well as the obligately paedomorphic sirenids engage in dimensionally complex chewing (non‐mammalian mastication) (Richard et al. 2023; Schwarz, Konow, Roba, et al. 2020; Schwarz et al. 2026). Later larval and paedomorphic forms often engage in uniarcuate chewing (Schwarz, Konow, Porro, et al. 2020; Spence et al. 2023), while metamorphic salamanders engage in tongue‐palate rasping (Heiss et al. 2019; Schwarz, Konow, Porro, et al. 2020; Schwarz et al. 2023). Finally, Japanese giant salamanders engage in a unique processing mechanism involving arcuate chewing combined with hemimandibular long‐axis rotation (Matsumoto et al. 2024).

Most studies on salamander feeding have characterised jaw and tongue movements using either high‐speed video or videofluoroscopy (see references above), measured bite force (Deban and Richardson 2017), and quantified jaw and tongue muscle activity using electromyography (Reilly and Lauder 1991; Wainwright et al. 1989). Compared to other terrestrial vertebrates, there is scarce data on the internal mechanical response of the skull (deformation, strain, and stress) during feeding in salamanders. To our knowledge, skull strains during feeding have never been directly measured in salamanders as, for example, in lizards (Porro et al. 2014; Ross et al. 2018). This may be due to the small size of many salamanders and the thinness of their skull bones, which makes implanting strain gauges difficult. Finite element analysis (FEA) is a modelling technique that predicts stress, strain and deformation in structures under loads. Over the past 20 years, FEA has been widely applied to address questions of organismal form and function, particularly in vertebrate skulls (Rayfield 2007). By predicting stress and strain within the skull under loads, FEA can be used to test hypotheses of function and understand the impact of feeding in driving skeletal evolution (Currey 2002; Rayfield 2007). Unlike strain gauges, FEA is non‐invasive and can predict mechanical response across the entire skull, rather than being constrained to areas of accessible bone. Although FEA has been applied to a wide range of living and fossil reptile and mammal skulls, there have been few studies using FEA to investigate skull mechanics in amphibians, with only three studies focused on salamander skulls (Fortuny et al. 2015, 2016; Zhou et al. 2017) and additional work on the skulls of temnospondyls (Fortuny et al. 2011, 2016; Konietzko‐Meier et al. 2018; Lautenschlager et al. 2016; Marcé‐Nogué et al. 2015), considered by many workers to be the ancestors of lissamphibians. Nonetheless, compared to other tetrapod groups, our understanding of the internal mechanical response of the salamander skull under feeding loads is limited.

This study aimed to address gaps in existing research by comparing skull anatomy, jaw musculature, and skull mechanics in two species of salamanders—the fully aquatic neotenic *Necturus maculosus* (the mudpuppy) and the metamorphic and terrestrial *Salamandra salamandra* (the fire salamander)—through micro‐computed tomography (µCT) scanning, 3D visualisation, physical and digital dissection, and FEA. We used these taxa and techniques to test five hypotheses:

We predict that *S. salamandra* will have relatively larger m. levator mandibulae muscles, used for forceful biting and food processing on land, whereas *N. maculosus* will have relatively larger m. depressor mandibulae muscles, allowing for fast jaw opening during suction feeding in water.

We predict that *S. salamandra* will have higher mechanical advantage of the jaw‐closing muscles for biting, whereas *N. maculosus*'s need to move the jaw quickly during suction feeding will be associated with lower mechanical advantage of the jaw‐closing muscles.

We predict that the skull of *S. salamandra* will experience lower peak stress during biting than that of *N. maculosus*, suggesting increased strength for resisting bite loads.

We predict that the skull of *S. salamandra* will exhibit more evenly distributed stress under bite loads, reflecting increased adaptation of skull morphology to biting compared to *N. maculosus*.

We predict that the presence of sutures will reduce peak stress magnitudes in both species compared to models without sutures.

## 2 Materials and Methods

### 2.1 Scanning, Staining, Image Processing and Model Preparation

This study utilised one adult specimen each of *Necturus maculosus* (Rafinesque 1819; basal skull length [BSL] 34.31 mm) and *Salamandra salamandra* (Linnaeus 1758; BSL: 18.17 mm); other specimen measurements are presented in Table 1. Specimens were obtained from collaborators (*N. maculosus* from Egon Heiss, formerly at Frederich‐Schiller University, Jena, Germany; *S. salamandra* from John Hutchinson, Royal Veterinary College) from unrelated previous studies. We aimed to select adults of one fully aquatic and one terrestrial taxon; the precise choice of species was due to specimens available from previous studies. *N. maculosus* was fixed in formalin while *S. salamandra* was originally frozen and later fixed in 4% paraformaldehyde in a phosphate buffered saline. The specimens were scanned at the University of Bristol School of Life Sciences on an X‐Tek H 225 µCT scanner, in a background medium of air with a tungsten target to produce stacks of 16‐bit TIFFs. *S. salamandra* was scanned at 135 kV and 375 uA, with no filter, at a resolution of 0.05065 mm/voxel. *N. maculosus* was scanned at 120 kV and 195 uA, with no filter, at a resolution of 0.02796 mm/voxel.

**Table 1:** Key head measurements from both taxa taken from Amira. Basal skull length is from the anterior tip of the upper jaw to the posterior end of the occiput. Head width is taken at the mediolaterally widest point. Height is measured as the vertical distance from skull roof to the level of the bottom of the quadrates. Tooth row length is for the lower jaw; for *N. maculosus*, lower jaw tooth row length includes the coronoid teeth, which extend posterior to the end of the dentary tooth row.

Measurement	*N. maculosus*	*S. salamandra*
Basal skull length (mm)	34.31	18.17
Head width (mm)	28.41	18.6
Head height (mm)	9.38	6.72
Preorbital length (mm)	9.10	7.84
Mandibular length (mm)	24.26	18.26

After initial scanning, specimens were prepared for diffusible iodine‐based contrast‐enhanced µCT (diceCT) to visualize soft tissues in CT scans (Gignac et al. 2016). The *S. salamandra* head was stained in 5% I_2_KI for 4 days before being re‐scanned at 160 kV and 188 uA at a resolution of 0.01547 mm/voxel. The head of the *N. maculosus* specimen was stained in 5.5% I_2_KI for 16 days and scanned at 160 kV and 150 uA at a resolution of 0.03202 mm/voxel. After scanning, the specimens were gradually transferred to 70% ethanol for long‐term storage. µCT data for both the unstained and stained specimens are deposited in MorphoSource and are freely available.

All scan data were segmented in Avizo 9.4 Lite (Thermo Fisher Scientific, Waltham, USA). In unstained CT scans, thresholding was used to separate high‐density bones from lower‐density tissues. Segmentations were refined using the paintbrush tool and interpolation to separate individual bones and sutures from each other and produce 3D surface models of individual bones. Existing anatomical descriptions were used to identify bones and soft tissues in the scan data (Francis 1934; Kleinteich et al. 2014). Care was taken to ensure a minimum three‐voxel thickness was maintained for each suture in all three orientations, completely separating adjacent bones. The resulting surface models were simplified if over 500,000 faces (the *N. maculosus* upper jaw was simplified to 359,707 faces, the lower jaw was simplified to 103,213 faces; the *S. salamandra* upper jaw was simplified to 357,091 faces, the lower jaw left at 410,332 faces), resulting in lower resolutions but more feasible meshes for running FEA. The models were moved relative to the global axes so that the origin is situated midway between the two jaw joints, the *y*‐axis was aligned dorsoventrally (upwards being positive), the x‐axis was aligned mediolaterally (left being positive) and passed through both jaw joints, and the *z*‐axis was aligned anteroposteriorly (anterior is positive) along the sagittal midline. The lower jaws were rotated to a gape angle of 16° for both taxa, the average gape angle during feeding in terrestrial and aquatic alpine newts (Heiss et al. 2018). Surface files for individual bones were exported as Drawing Interchange Format (DXF) files for FEA (see below).

Stained CT scans were segmented in the same manner, using paintbrush and interpolation tools (interpolating across no more than five slices) to separate individual jaw muscles from each other. Muscle attachment sites on the skull were initially identified from diceCT data and mapped onto the models in Avizo, then exported as STL files. The XYZ coordinates of muscle attachment site centroids were identified using the MATLAB script Area_Centroids>From_ STL (Davis et al. 2010; Santana et al. 2010). Muscle volumes and centroid‐centroid muscle lengths were determined using measurement tools in Avizo. Full details of measurements taken in Avizo are available in Supporting Table 1.

### 2.2 Physical Dissection

Specimens were physically dissected after diceCT. All measurements were taken using digital callipers with an accuracy of 0.01 mm and an electronic balance sensitive to 0.0001 g. Physical dissection confirmed attachment sites identified in µCT data, including confirming tendinous attachment sites, and muscle weights and lengths were obtained. The following muscles were dissected in both specimens: m. levator mandibulae externus, m. levator mandibulae longus, m. levator mandibulae internus, m. depressor mandibulae, and m. depressor mandibulae posterior. In both specimens, the two depressor muscle subdivisions could not be physically separated and were dissected and weighed together, although they could be visualised separately in diceCT data. Both species exhibit complex hyoid musculature that can be visualized in diceCT data; however, because the musculature is incomplete (due to where the heads were severed) and not used for FEA, they are not described or illustrated in this study.

Muscle fibre lengths were measured both prior to and after removal of each muscle; three fibres were measured to obtain an average length. During muscle removal, the locations and nature of the origins and insertions were recorded to check muscle attachment sites identified from diceCT data before mapping attachment sites onto finite element models (FEMs). For muscles exhibiting pennation, the total muscle length, distal tendon length, muscle fibre length and the perpendicular distance from the tendon to the edge of the muscle were recorded, following existing protocols (Anapol and Barry 1996; Taylor and Vinyard 2004). Muscles were weighed and stored in 70% ethanol. All muscle measurements taken during the dissection are recorded in Supporting Tables 2 and 3.

### 2.3 Calculating Muscle and Bite Forces

To calculate muscle forces, mechanical advantage, and bite forces, we averaged left and right muscle volumes and total muscle lengths (centroid to centroid distance) taken in Avizo. However, for pennate muscles, we used muscle volumes from digital dissections but fibre lengths and pennation angles measured in physical dissections. See Table 2 for values used in the calculations and applied to the models (full details are given in Supporting Tables). For muscle volumes (from digital dissections), we present the percentage of total muscle mass represented by individual muscles in Table 2, allowing us to test H1. Muscle force for parallel‐fibred muscles was estimated using Equation (1) (Sacks and Roy 1982):

$$\mathrm{Force}=\frac{\mathrm{Volume}}{\mathrm{Lm}}\times \mathrm{Specific}\;\mathrm{tension}$$

**Table 2:** Table of muscle measurements used to calculate muscle force. The left‐side and right‐side volume and length were averaged for each muscle. Muscle volume and lengths were measured from digital dissections in Avizo while pennation angle and fibre lengths of pennated muscles were measured in the physical dissection.

	Levator internus	Levator externus	Levator longus	Depressor	Depressor posterior	Total muscle volume
***Necturus***						
Average volume (mm^3^)	503.72	682.52	253.32	90.91	276.57	1807.04
% of total muscle volume	27.88	37.77	14.02	5.03	15.30	
Average length (mm)	25.91	23.12	24.16	17.07	22.21	
Pennation angle (°)	27.06	none	none	none	14.86	
Fibre lengths (mm)	8.64	none	none	none	7.68	
PCSA (mm^2^)	51.92	29.53	10.49	5.33	34.81	
Force (N)	12.98	7.38	2.62	1.33	8.70	
***Salamandra***						
Average volume (mm^3^)	7.43	8.99	11.51	2.67	3.56	34.16
% of total muscle volume	21.74	26.31	33.71	7.81	10.43	
Average length (mm)	6.22	5.95	11.60	6.68	6.68	
PCSA (mm^2^)	1.19	1.51	0.99	0.40	0.53	
Force (N)	0.30	0.38	0.25	0.10	0.13	
***Sal.* (rescaled)**						
Average volume (mm^3^)	26.60	32.19	41.24	9.56	12.75	122.34
% of total muscle volume	21.74	26.31	33.71	7.81	10.43	
Average length (mm)	9.52	9.10	17.75	10.22	10.22	
PCSA (mm^2^)	2.79	3.54	2.32	0.94	1.25	
Force (N)	0.70	0.88	0.58	0.23	0.31	

In which *Lm* is muscle length and specific tension of vertebrate striated muscle is 25 N cm^**−**2^ (Rospars and Meyer‐Vernet 2016). For pennate muscles, force was estimated using Equation (2) (Anapol and Barry 1996):

$$\mathrm{Force}=\frac{\mathrm{Volume}\times \cos\theta }{\mathrm{Lf}}\times \mathrm{Specific}\;\mathrm{tension}$$

in which *Lf* is muscle fibre length and *θ* is the pennation angle of the muscles (Murphy and Beardsley 1974; Anapol and Barry 1996). For both taxa, we assumed simultaneous and maximal muscle contraction.

3D moment arm lengths were calculated for the muscles on the left side of the skull using the *XYZ* coordinates of the centroids of the left side muscle origins and insertions, and of the left side jaw joint. Bite forces were estimated at three different points for each taxon: we selected the tooth on the left upper jaw closest to an anterior (~10%), middle (~50%), and posterior (~90%) point measured from the front of the tooth row. 3D distances were measured from the jaw joint to these teeth in Avizo.

Bite force was calculated using Equation (3) (Lautenschlager 2013):

$$\mathrm{Bite}\;\mathrm{Force}=\frac{(\sum \mathrm{Muscle}\;\mathrm{Forces}*\mathrm{Muscle}\;\mathrm{Moment}\;\mathrm{Arms})}{\mathrm{Bite}\;\mathrm{Point}\;\mathrm{Moment}\;\mathrm{Arm}}$$

The calculated unilateral bite force was divided by the input muscle forces (excluding the depressors) to estimate mechanical advantage, allowing us to test H2.

As *N. maculosus* is substantially larger than *S. salamandra*, we compared muscle volumes, cross‐sectional areas and forces with the skull of *S. salamandra* scaled to the same skull width as that of *N. maculosus*, following findings in frogs (Lappin et al. 2017) and chameleons (Measey et al. 2009) that bite force is best predicted by skull width. This allowed us to look at the impact of skull shape on bite force independent of skull size.

### 2.4 Finite Element Analysis

FEA was carried out in Strand7 (Strand7 Pty Ltd., Sydney, Australia). DXF surface files of individual bones and sutures were imported into Strand7, free edges were automatically repaired, and *t*‐junctions were manually removed. The cleaned models were meshed using four‐node (linear) tetrahedral elements. The model of *N. maculosus* consisted of 1,342,086 elements in the upper jaw, and 575,321 elements in the lower jaw; the model of *S. salamandra* consisted of 1,596,770 elements in the upper jaw, and 412,668 elements in the lower jaw. The number of elements in all models were above thresholds necessary for convergence (McCurry et al. 2015; Tseng et al. 2011). Although it is likely these taxa employ different jaw mechanisms to capture and process food and feature different material bone and soft tissue material properties, to enable direct comparison between the species we modelled both skulls under simple, orthal jaw closure using the same material properties. The jaw joints were constrained at two nodes per articulation surface and allowed to rotate around the x‐axes to permit rotation about the joint axis. Six bite points were analysed for each taxon: anterior, middle and posterior unilateral bite points on the left side of the head, as well as anterior, middle and posterior bilateral bites—see previous section for how bite points on the upper jaw were determined. For FEMs we also constrained the closest opposing tooth on the lower jaw. The tip of the tooth closest to these locations was fixed in all degrees of freedom. As no data is currently published on the material properties of salamander skull bone or suture, we assigned bones in both models the isotropic material properties of alligator bone (Young's modulus: 17,000 MPa; Poisson's ratio: 0.3; density: 1 × 10^−6 ^kg/mm^3^; Zapata et al. 2010), and sutures, including the mandibular symphysis, were assigned isotropic material properties of alligator sutures (Young's modulus: 90 MPa; Poisson's ratio: 0.3; density: 1 × 10^−6 ^kg/mm^3^; Porro et al. 2011).

The models were loaded using the MATLAB script Boneload (Grosse et al. 2007), to account for the curvature of muscles around the skull and evenly distribute muscle forces onto bricks within attachment sites identified in the stained µCT dataset. Only forces from the jaw‐closing muscles were applied to FEMs. For *S. salamandra*, the m. levator mandibulae longus and externus share the longus' origin on the dorsal aspect of the skull. The closest node to the centroids of muscle origins and insertions (see previous) was identified on the FEMs and used as the focal point at which to ‘aim’ the muscle forces in Boneload.

We analysed three sets of models: one set of all six bite scenarios with muscle forces at ‘life size’; one set of all bite scenarios with the model of *S. salamandra* scaled to the same surface area: force ratio of *N. maculosus* to remove the effects of differences in taxon size and provide a comparison of von Mises stress performance (a proxy for strength) based solely on shape (Dumont et al. 2009); and a single model of each taxon under life size muscle loads during a middle unilateral bite with suture elements given the material properties of bone (i.e., a solid model). Linear static solutions were performed for all crania and mandibles in Strand7. Performance metrics examined include bite forces (Y/dorsoventral only) generated at constrained teeth, peak stress magnitudes (after removing the top 1% of values to account for high point stresses at constraints and modelling artifacts, allowing us to test H3), and contour plots of stress (allowing us to test H4). We examined von Mises stress, which represents a measure of overall stress with higher magnitudes indicating closer proximity to failure and a mechanically weaker structure under the load being tested (Dumont et al. 2009; Rayfield 2007). Comparison of scaled models with and without sutures during middle bites allowed us to test H5.

**Figure 1:**
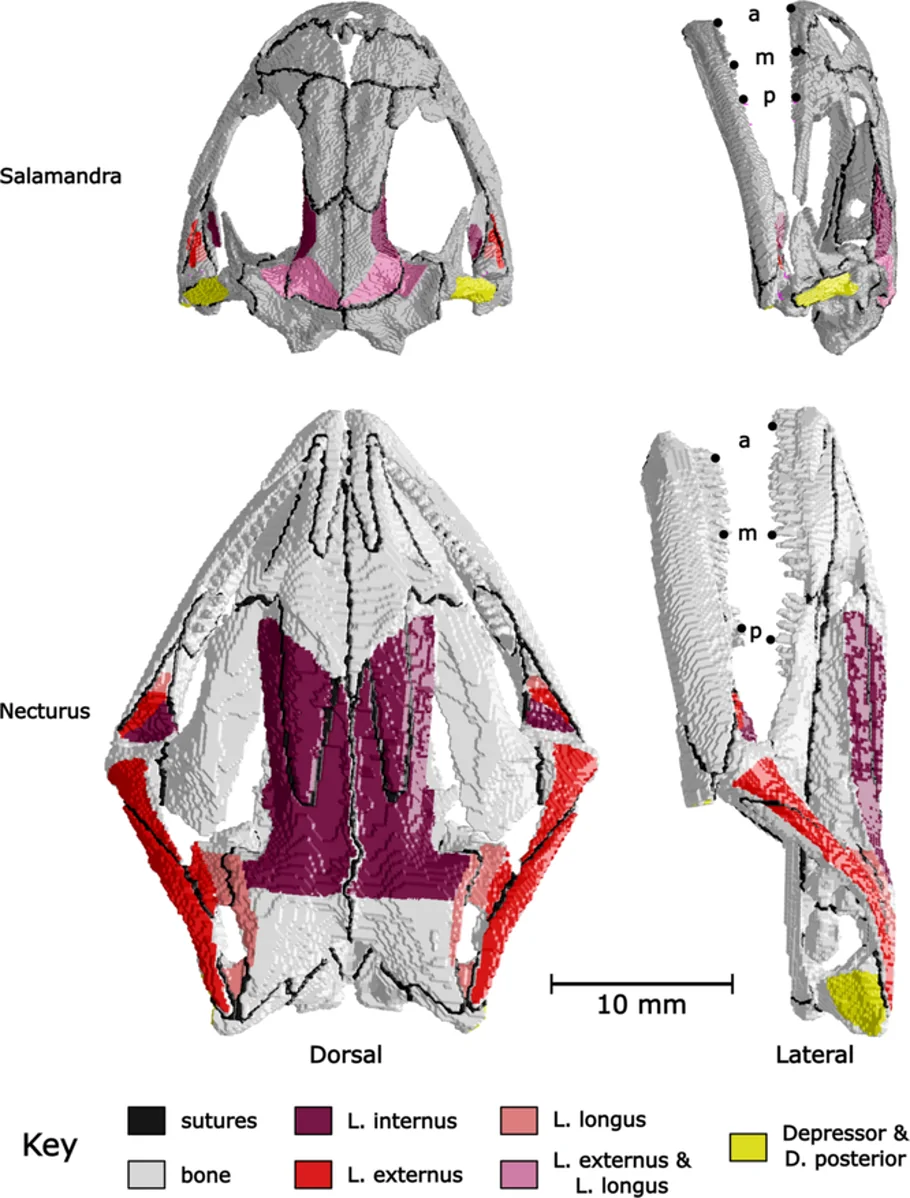
Mapped muscle attachment surfaces and bite points in Strand7 for the FE models. Anterior (a), middle (m) and posterior (p) bite points are shown, and muscles that originate from the fascia of another muscle are grouped with that muscle as a single attachment (e.g., the combined m. levator mandibulae externus and longus origins in *S. salamandra*).

The mapped muscle attachments on the loaded FE meshes are shown in Figure 1, and the constraints of the unilateral (left‐side) anterior, middle, and posterior bite points are all marked in lateral view.

## 3 Results

### 3.1 Skeletal Anatomy

The skull of *N. maculosus* was 1.9 times longer than the skull of *S. salamandra* (Table 1). The skull of *N. maculosus* was relatively narrower (0.83 width: length ratio) compared to *S. salamandra* (1.02), dorsoventrally flatter (0.27 height: length ratio) than *S. salamandra* (0.37), and had a substantially shorter pre‐orbital region (27% skull length in *N. maculosus* compared to 43% *S. salamandra*). For both taxa, the dentary tooth row extended just over half the length of the lower jaw (0.55 in *N. maculosus* and 0.51 in *S. salamandra*). However, the lower jaw of *N. maculosus* was 71% the length of the upper jaw, while in *S. salamandra* the lower jaw was slightly longer (100.5%) than the upper jaw; as a result, the tooth row was proportionately longer compared to basal skull length in *S. salamandra* (0.52) than in *N. maculosus* (0.39). The external nares of *S. salamandra* were relatively much larger than those of *N. maculosus*, and *S. salamandra* features a prominent midline gap (the cavum internasale, *sensu* Villa et al. 2014) between the left and right nasals and premaxillae. In summary, *N. maculosus*'s head was proportionally flatter and narrower than *S. salamandra*'s, with a shorter snout and an extended postorbital region, as well as a proportionally shorter lower jaw.

**Figure 2:**
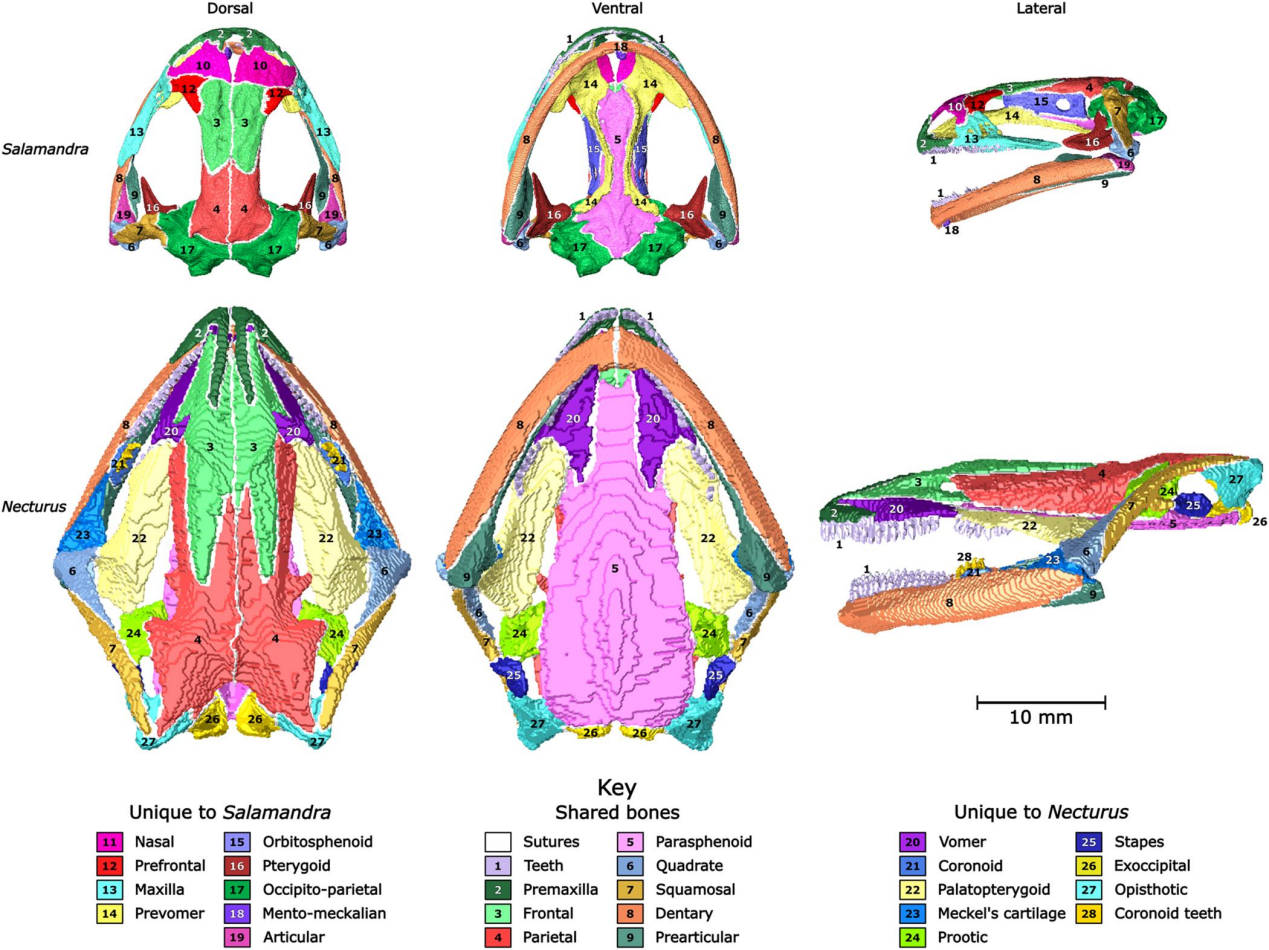
Segmented 3D models of both taxa, with bones assigned different colours.

The 3D models of the segmented skulls are shown in Figure 2, with individual bones colour‐coded. The two skulls exhibited substantial morphological differences. *N. maculosus* possessed a large palatopterygoid as well as the vomer, coronoid, prootic, stapes, exoccipital, and opisthotic bones that were absent in *S. salamandra*, while *S. salamandra* possesses prevomer, nasal, prefrontal, maxilla, orbitosphenoid, pterygoid, occipito‐parietal, and mento‐Meckelian bones that were absent in *N. maculosus*. There were also notable differences in some bones present in both species—for example, in *S. salamandra* the premaxilla was a single fused bone but it was separated into left and right sides in *N. maculosus*.

The two taxa also exhibited notable differences in dentition. *S. salamandra* had 96 marginal teeth in the upper jaw (premaxilla and maxilla) and 44 teeth in the lower jaw (dentary), with a large number of teeth on the roof of the mouth (prevomer) as is common among metamorphosed salamanders (Trueb 1993); palatal teeth could not be counted due to their very small size. *N. maculosus* had 60 teeth in the upper jaw (11 [left] and 12 [right] premaxilla, 12 [left] and 11 [right] vomer and 7 [left and right] palatopterygoid) and 41 teeth in the lower jaw (18 [left] and 16 [right] dentary and 3 [left] and 4 [right] coronoid), all of which were large enough to be segmented.

In *S. salamandra*, the jaw joint was formed between the quadrate and ossified articular, the latter featuring complex internal cavities visible in µCT scans. In *N. maculosus* the jaw joint was formed between the quadrate and the posterior part of the Meckel's cartilage; when ossified, this portion forms the articular. The jaw joint of *N. maculosus* was strongly anteriorly displaced, compared to its more posterior placement in *S. salamandra*.

### 3.2 Muscular Anatomy

**Figure 3:**
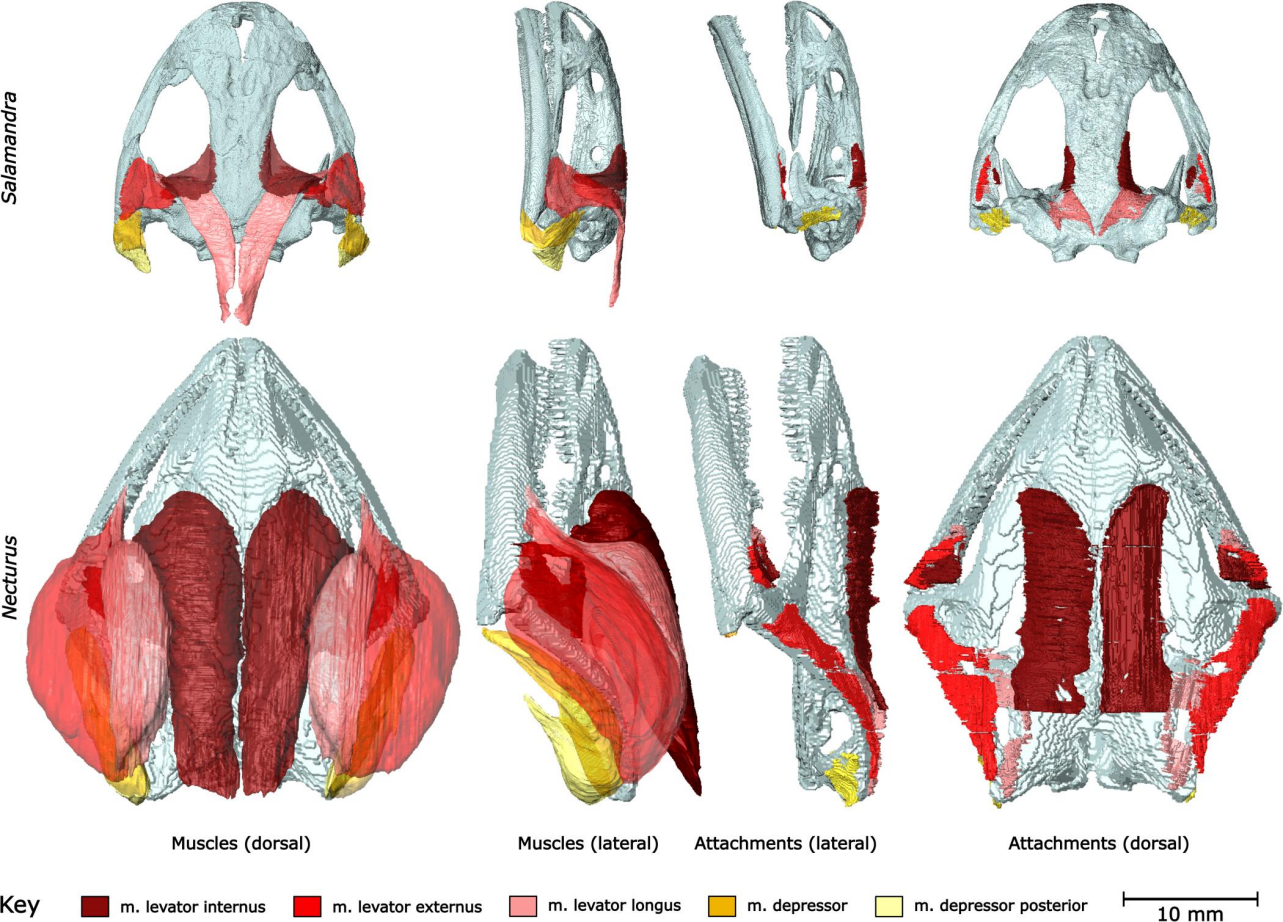
3D digital dissections of the jaw adductor and depressor muscles (left two columns) and attachments sites (right two columns) from diceCT data in dorsal (far left and far right columns) and left lateral (central columns) views.

The nomenclature here follows Francis (1934), which contained high‐quality plates of dissections we used as a point of comparison when naming specific structures. More recent nomenclature would refer to the levator muscles as the adductor muscles, and the levator longus as the adductor posterior. The jaw opening (m. depressor mandibulae muscles) and closing muscles (m. levator mandibulae muscles) were subdivided into distinct portions in both taxa: the m. levator mandibulae externus, m. levator mandibulae longus and m. levator mandibulae internus, and the m. depressor mandibulae and m. depressor mandibulae posterior muscles (Figure 3). In *N. maculosus*, the unipennate fan‐shaped m. levator mandibulae externus originated on the anterodorsal surfaces of the squamosal and quadrate, and on the prootic, and inserted via an aponeurosis on the dorsolateral aspect of the posterior dentary and Meckel's cartilage. The fusiform, parallel fibred m. levator mandibulae longus originated on the dorsal aspects of the opisthotic, parietal and prootic, and curved ventrally to insert via an aponeurosis on the dorsal aspect of the dentary, immediately anterior to the insertion of m. mandibulae levator internus. The unipennate m. levator mandibulae internus had a broad origin on the dorsal aspects of the parietal and frontal (with the most posterior fibres attaching to the fascia overlying the epaxial muscles, not to the skull itself) and inserted via a tendon on the dorsal aspect of the Meckel's cartilage. The m. depressor mandibulae and m. depressor mandibulae posterior of *N. maculosus* were fused, with a shared origin on the lateral face of the opisthotic bone, and a shared insertion on the Meckel's cartilage. The m. depressor mandibulae was a parallel‐fibred muscle, and the m. depressor mandibulae posterior was a unipennate muscle with a tendinous insertion.

In *S. salamandra*, the parallel‐fibred m. levator mandibulae externus originated from the fascia dorsally overlying the m. levator mandibulae longus and inserted on the dorsal aspects of the articular and dentary, lateral to the insertions of the other jaw‐closing muscles (Figure 3). The muscle was closely associated with a blood vessel and nerve, thought to be the arteria mandibularis internus and nervus trigeminus ramus mandibularis (Francis 1934), that course between the m. levator mandibulae externus and m. levator mandibulae longus. The parallel‐fibred m. levator mandibulae longus originated on the dorsal aspects of the occipitoparietal and parietal, and curved sharply to insert via a tendon on the dorsal aspect of the articular in *S. salamandra*, between the insertions of m. levator mandibulae externus and internus. The parallel‐fibred m. levator mandibulae internus originated on the dorsolateral aspect of the parietal and inserted via an aponeurosis on the dorsal aspects of the articular and prearticular, medial to the insertion of m. levator mandibulae longus. As in *N. maculosus*, the parallel‐fibred m. depressor mandibulae and m. depressor mandibulae posterior of *S. salamandra* were fused and could not be physically separated. They had a shared origin on the posterior aspects of the quadrate and squamosal and an aponeurotic insertion on the posterior articular. Both muscles curved around the cartilaginous skeleton of the hyoid apparatus. Some fibres of the m. depressor mandibulae posterior may merge with the epaxial muscles, but detail was difficult to see even under a microscope.

All of the jaw muscles were substantially larger in *N. maculosus* than in *S. salamandra*, both in terms of absolute and scaled size (Table 2). Even when scaled to the same head width, the combined volume of the jaw elevator muscles in *N. maculosus* was over 14 times the size of those in *S. salamandra*, while the combined volume of both depressors in *N. maculosus* was 16.5 times larger than those of *S. salamandra*. Pennation of two muscles (m. levator mandibulae internus and m. depressor mandibular posterior) in *N. maculosus* resulted in even greater discrepancy in muscle force between the two taxa (Table 2). Interestingly, in terms of the relative contribution of each muscle, the total contribution of the depressors to muscle volume in *N. maculosus* (20.33%) was only slightly greater than in *S. salamandra* (18.24%). Instead, in terms of relative contributions, the m. levators mandibulae internus and externus were proportionately larger in *N. maculosus* while m. levator mandibulae longus was proportionately larger in *S. salamandra*.

Thus, in terms of absolute and scaled size, the jaw‐closing muscles of *S. salamandra* were smaller than those of *N. maculosus*, while differences between the taxa in terms of the relative contributions of the jaw opening and closing muscles were minimal, contrary to the predictions of H1.

### 3.3 Estimated Bite Forces and Mechanical Advantage

Table 3 shows calculated bite force and mechanical advantage at anterior, middle and posterior bite points for both taxa, including both the life size and scaled data from *S. salamandra*. In both taxa, bite force and mechanical advantage increased as bite point moved posteriorly, as expected. Estimated bite forces were 22.3–34.3 times greater in *N. maculosus* at all bite points at life size and 9.6–14.8 times when scaled to the same head width, reflecting the much larger jaw‐closing muscles of *N. maculosus* (see above). *S. salamandra* featured marginally higher mechanical advantage than *N. maculosus* during biting at the anterior end of the jaw; however, *N. maculosus* featured substantially higher mechanical advantage during posterior bites. Thus, H2 was only partially supported.

**Table 3:** Table of calculated bite forces and mechanical advantages (output bite force: input muscle force) at all tested bite points, for both taxa and the rescaled Salamandra model.

	Anterior	Middle	Posterior
*Necturus* bite force (N)	3.13	4.48	7.72
*Salamandra* bite force (N)	0.14	0.17	0.23
*Sal*. rescaled bite force (N)	0.33	0.39	0.52
*Necturus* mechanical advantage	0.14	0.20	0.34
*Salamandra* mechanical advantage	0.15	0.18	0.24
*Sal*. rescaled mechanical advantage	0.15	0.18	0.24

### 3.4 Finite Element Analysis

In both taxa, bite force increased as bite point moved posteriorly during both unilateral and bilateral biting, with summed forces from both sides of the head during bilateral bites being marginally higher than forces recorded at single teeth during unilateral bites (Table 4). Unilateral bite forces recorded from FEMs were similar to those predicted by 3D lever arm mechanics, being slightly higher at all bite points in *N. maculosus*, and slightly higher during anterior and middle bites but lower during posterior bites in *S. salamandra*.

**Table 4:** Table of peak von Mises stress and bite forces for all the tested bite points, for both taxa and the rescaled Salamandra model. For the stresses, values from the upper (U) and lower (L) jaws are reported.

		Unilateral anterior	Unilateral middle	Unilateral posterior	Bilateral anterior	Bilateral middle	Bilateral posterior
*Necturus* peak stress (MPa)	U	11.52	10.33	21.73	9.72	8.59	14.29
	L	16.17	15.99	18.38	15.75	14.81	13.71
*Salamandra* peak stress (MPa)	U	1.73	1.71	2.18	1.59	1.28	1.79
	L	3.36	3.14	2.80	3.07	2.91	2.63
*Sal*. rescaled peak stress (MPa)	U	17.54	17.35	22.07	16.15	12.96	16.71
	L	29.67	27.66	24.68	27.07	25.69	23.17
*Necturus* bite force (N)		4.168	6.182	8.427	4.326	6.373	9.961
*Salamandra* bite force (N)		0.181	0.199	0.211	0.185	0.225	0.255
*Sal*. rescaled bite force (N)		1.834	2.01	2.133	1.870	2.271	2.578

We observed some similar trends in peak von Mises stress values in both life‐size models (Table 4). For a given bite point, stress was always lower in the upper jaw than in the lower jaw in *S. salamandra*. This was also true for *N. maculosus* except during both unilateral and bilateral posterior bites, in which peak stress in the cranium exceeded that in the mandible. For both taxa, peak stresses in both the upper and lower jaw were always higher during unilateral bites than during bilateral bites at the same tooth position, reflecting increased torsion of the cranium and mandible during unilateral biting. For both taxa, the lowest peak stresses in the upper jaw occurred during middle bites (both uni‐ and bilateral), with the highest peak stresses during posterior bites. In contrast, the lower jaws exhibited different trends in peak stresses both between the two taxa and during uni‐ versus bilateral biting: in *S. salamandra*, peak stress was highest during anterior bites and decreased as bite point moved posteriorly for both uni‐ and bilateral bites. In *N. maculosus*, in contrast, during unilateral biting peak stress in the lower jaw was lowest during middle bites and highest during posterior bites, as in the upper jaw. However, during bilateral bites, peak stress in the lower jaw of *N. maculosus* was highest during anterior bites and decreased as bite point moved posteriorly. Under all loading conditions, the life‐size model of *S. salamandra* exhibited substantially lower stress than that of *N. maculosus*, reflecting its much smaller jaw‐closing muscles.

During anterior biting (both unilateral and bilateral) in *N. maculosus*, the premaxilla, squamosals and quadrates were deflected dorsally and anteriorly while the remainder of the upper jaw was deformed downwards by the action of the jaw‐closing muscles. During middle and posterior unilateral bites, the upper jaw anterior to the level of the prootic was deformed downwards with the snout twisting about its long axis so that the working side rotates dorsally and the balancing side rotates ventrally. The posterior part of the braincase as well as the quadrates and squamosals deformed dorsally and anteriorly. Deformation of the upper jaw was most pronounced during posterior unilateral bites. During middle and posterior bilateral bites, the upper jaw deformed symmetrically, with the anterior snout and palate being bent downwards and the parietals, squamosals and quadrates deflected upwards and anteriorly. During anterior unilateral biting, the working side mandible of *N. maculosus* was bent upwards and slightly medially; in contrast, the balancing side was deformed to a greater extent, being bent upwards, medially, and the dorsal margin twisted inwards. The mandible deformed in the same way during middle and posterior unilateral biting, but deformation became more pronounced as bite point moved posteriorly, with the symphysis being deformed towards the working side. During bilateral biting, both mandibular rami were bent upwards and medially, with deformation most pronounced during anterior bites.

During anterior unilateral and bilateral bites, the anterior snout of *S. salamandra* bent upwards, the posterior skull roof and palate were deflected downwards, and both maxillae splayed laterally. During middle and posterior unilateral bites, the anterior snout was bent downwards and the entire upper jaw twisted about its long axis so that the working side deformed upwards and the balancing side deformed downwards, with deformation being more pronounced during posterior bites. During middle and posterior bilateral bites, deformation of the cranium was symmetrical with the anterior snout, palate and braincase deformed downwards and the maxillae deflected upwards. During anterior unilateral and bilateral bites, the middle parts of both sides of the mandible of *S. salamandra* were pulled upwards and medially, and the symphyseal region was deflected downwards. During middle and posterior unilateral bites, the posterior and anterior sections of the working side mandible were bent upwards, whereas the entire balancing side ramus was bent dorsally and medially, the dorsal border twisted inwards, and the symphyseal region was twisted towards the working side. In comparison, during middle and posterior bilateral bites, the posterior parts of both mandibles were bent dorsally while the symphyseal region was deflected downwards.

**Figure 4:**
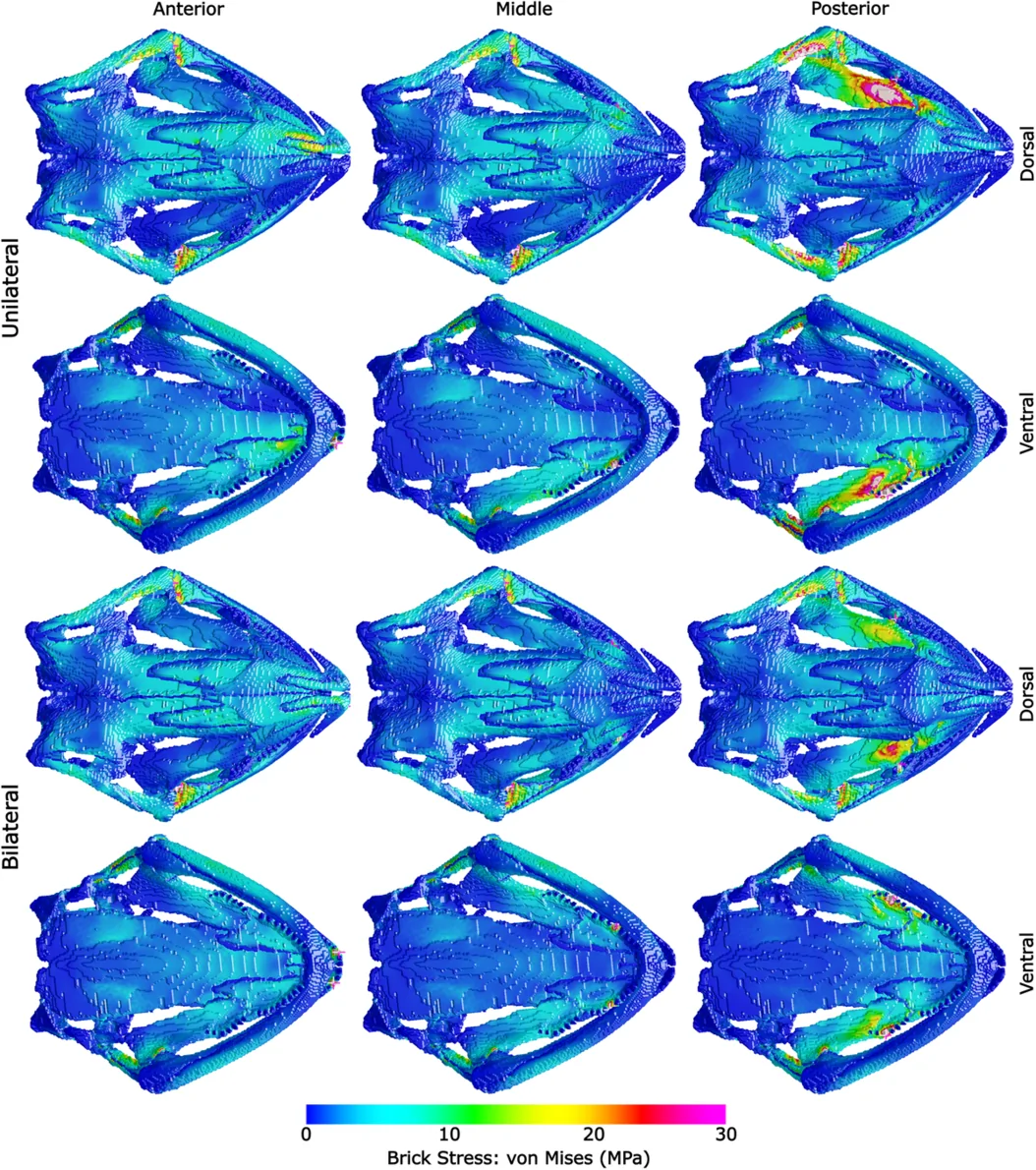
Dorsal and ventral views of von Mises stress in *N. maculosus* at all studied bites.

During all bites in *N. maculosus*, the squamosals, quadrates and adjacent part of the palatopterygoid experienced high stress, as do the posterior part of the frontals and anterior part of the parietals (Figure 4). During anterior unilateral and bilateral bites, high stress also occurred in the working side premaxilla, anterior frontal and vomer. During middle unilateral bites, high stress shifted to the working side vomer and palatopterygoid, while stress decreased in the balancing side palatopterygoid. Posterior unilateral bites generated large regions of high stress in the palatopterygoid. Stress distributions in the upper jaw were similar during middle and posterior bilateral bites as during unilateral biting but symmetrically distributed and lower in magnitude. During anterior unilateral bites, the lateral and medial aspects of the posterior lower jaw of *N. maculosus* experienced high stress, with higher stress in the balancing side ramus. As bite point moved posteriorly, the region of elevated stress spread anteriorly on the balancing side and a new region of high stress developed near the symphysis on the working side due to twisting of the symphysis. During anterior bilateral bites, both sides of the posterior portion of the lower jaw exhibited high stress. As bite point moved posteriorly, the region of high stress became more constricted at the posterior ends of the mandibular rami.

**Figure 5:**
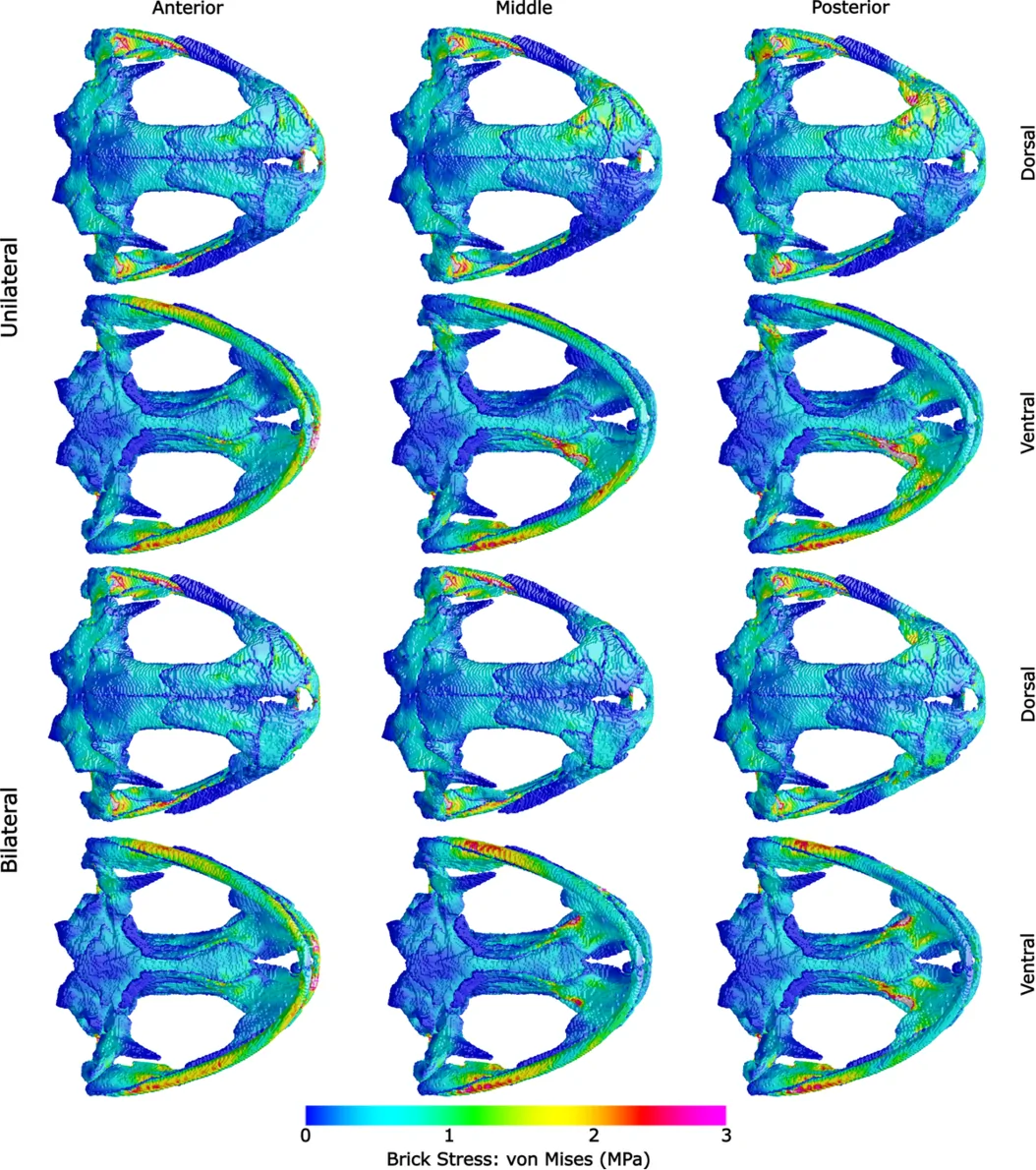
Dorsal and ventral views of von Mises stress in *S. salamandra* at all studied bites.

The squamosals and quadrates on both sides of the upper jaw of *S. salamandra* exhibited high stress during all bites without rescaling (Figure 5). During anterior unilateral bites there were areas of high stress in the working side premaxilla, prevomer, nasal and frontal. As the bite point moved posteriorly, stress increased on the working side and areas of high stress shifted posteriorly from the premaxilla and nasal to the maxilla and prefrontal. Areas of peak stress in the upper jaw during bilateral biting resembled those during unilateral biting except being symmetrical in distribution. The lower jaw of *S. salamandra* exhibited the highest stresses at the jaw joints, bite points and dorsal margin of the posterior dentary at all bite points. During anterior bites (both unilateral and bilateral), the posteroventral margins of both dentaries exhibited elevated stresses with another region of high stress near the anterior end of the dentaries. During unilateral middle and posterior bites, stress increased in the working side mandible, particularly posteroventrally. During middle and posterior bilateral bites, stress increased in the posterior part of the mandible and decreased at the symphysis.

**Figure 6:**
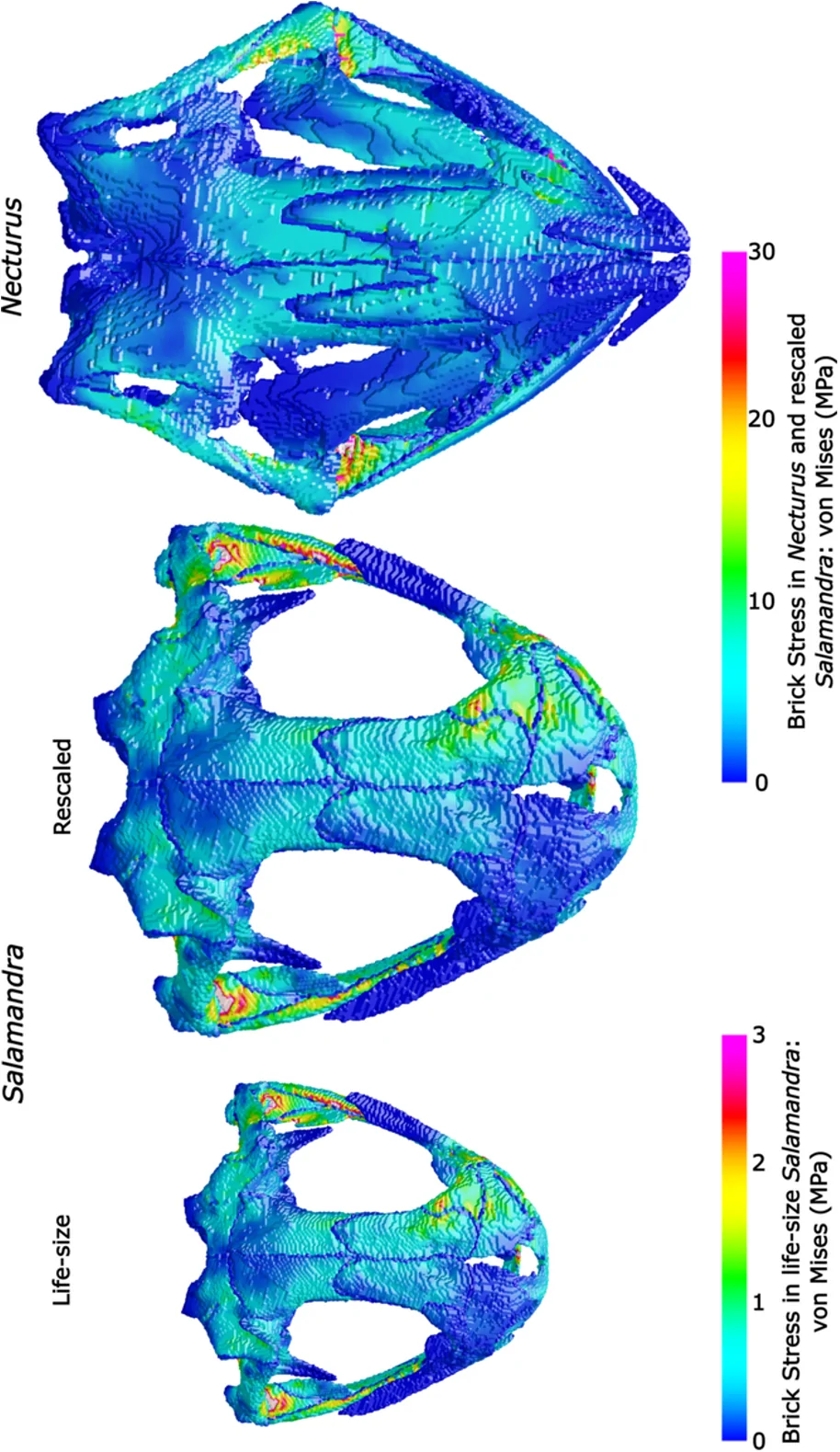
von Mises stress during unilateral biting at the middle of the tooth row in both taxa at life size, and a rescaled *S. salamandra*.

Scaling the model of *S. salamandra* to the same surface area: muscle force ratio as *N. maculosus* resulted in increased bite forces and peak von Mises stress magnitudes compared to the life size model (by approximately the same amount [x10] as the increase in muscle force applied to the scaled model). Trends in bite force and peak von Mises stress during unilateral middle biting in the scaled model were identical to those observed in the life size model. Likewise, stress distributions between the life size and scaled *S. salamandra* models were identical under the same loading conditions (Figure 6). Thus, as expected, increasing the muscle force applied to the *S. salamandra* model resulted in increased bite forces and peak stress magnitudes but did not change model behaviour. Compared to *N. maculosus*, the scaled model of *S. salamandra* exhibited substantial higher peak von Mises stresses under nearly all loading regimes, with peak stresses in the upper jaw 42% higher on average in the scaled model of *S. salamandra* compared to *N. maculosus*, and peak stresses in the lower jaw an average of 68% higher (Table 4). Only in one load case—posterior unilateral biting—did the upper jaw of *S. salamandra* exhibit only marginally higher (1%) peak stress than that of *N. maculosus*. These results suggested that the skull of *S. salamandra* is weaker compared to that of *N. maculosus* under similar loads, contrary to the predictions of H3. When contour plots of the scaled *S. salamandra* and the *N. maculosus* model were compared (Figure 6), both taxa exhibit areas of low stress (the posterior maxillae of *S. salamandra* and ventral braincases of both taxa). Overall, however, *S. salamandra* exhibited more homogeneously distributed stress, whereas *N. maculosus* exhibited pronounced patches of very high and very low stress, particularly in the lower jaw. This suggested that more of the skull is responding to and resisting feeding loads in *S. salamandra* than in *N. maculosus*, supporting H4.

**Figure 7:**
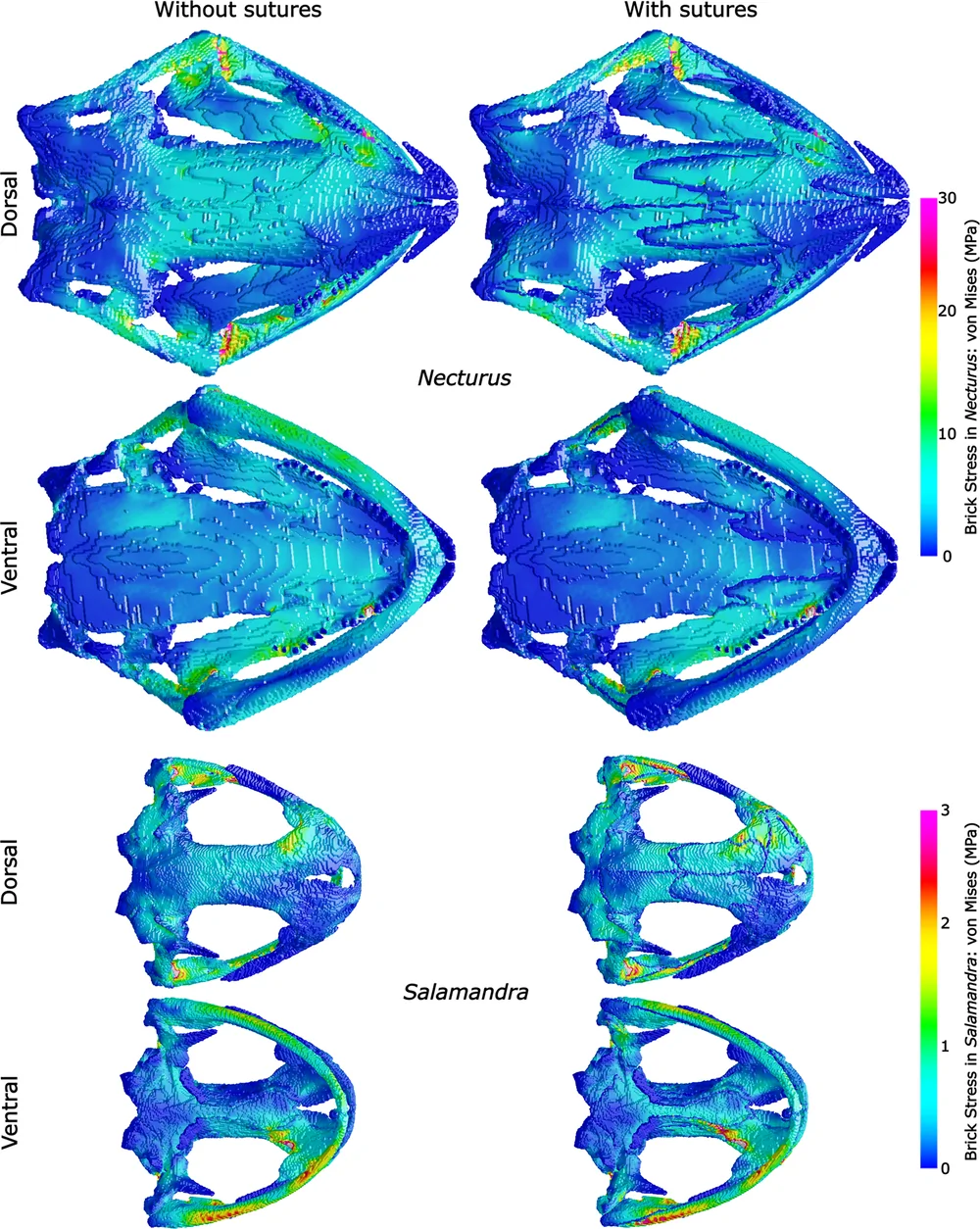
von Mises stress in both taxa at life size, where sutures have the properties of bone.

The impact of sutures on the mechanical behaviour of the skull was complex and varies between the two taxa. Bite force was 11% higher in the solid model of *S. salamandra* compared to the model containing sutures, but less than 1% higher in the solid model of *N. maculosus* compared to the suture model (Table 4). Peak von Mises stress was lower in solid models of *N. maculosus* (75% in the upper jaw, 73% in the lower jaw) and *S. salamandra* (77% in the upper jaw, and 89% in the lower jaw) compared to models with sutures (Table 4); these results were contrary to the predictions of H5 as including sutures led to increased (rather than decreased) peak stresses. In *N. maculosus*, overall stress distributions were similar between the solid and suture models (Figure 7), although areas of high stress on the squamosals, quadrates and articular were reduced in the solid model. However, new areas of high stress appeared at the palatopterygoid‐quadrate contact—especially on the balancing side—in the solid model compared to the suture model of *N. maculosus*. Stress distributions in the lower jaw of *N. maculosus* were nearly identical in the two models except that the coronoid bones and teeth, which were unstressed in the suture model, exhibited moderate stress in the solid model. As in *N. maculosus*, areas of very high stress on the squamosals, quadrates and articulars were reduced in the solid model of *S. salamandra* compared to the suture model. However, there were more pronounced differences in global stress patterns in the upper jaw of *S. salamandra*, with stress more constrained to the working side in the solid model compared to the suture model. Both models of *S. salamandra* featured areas of very high stress on the prefrontal and prevomer; however, areas of high stress in the dorsal part of the maxilla and at the nasal‐prefrontal contact in the suture models were substantially lower in the solid model. Most notably, in the *S. salamandra* model containing sutures, stress spread from the working side premaxilla to the balancing side. In contrast, in the solid model these stresses were more constrained to the working side. Stress distributions in the lower jaw of *S. salamandra* were nearly identical between the solid and suture models.

## 4 Discussion

This study combined high‐resolution imaging, 3D visualisation, dissection, and FEA to achieve several aims. In terms of osteology, we demonstrated substantial differences in the skulls of *S. salamandra* and *N. maculosus*, including distinct overall skull shapes, differences in the bones composing the skull, and variations in the distribution of the teeth. In particular, the skull of *N. maculosus* featured an elongate postorbital region and anteriorly displaced jaw joint compared to *S. salamandra*.

### 4.1 Multiple Factors Impact Relative Jaw Muscle Size and Mechanical Advantage

Because terrestrial taxa rely on biting with the jaws as well as tongue prehension to capture and ingest food—unlike aquatic taxa, which can employ suction feeding—we predicted (H1) that the terrestrial *S. salamandra* would have relatively larger jaw closing muscles than the aquatic *N. maculosus* (and that the latter would have relatively larger m. depressor mandibulae to produce strong jaw opening movements during suction feeding). Because levers generally exhibit a trade‐off between generating high force or high speed (Arnold et al. 2011), we further predicted (H2) that the skull of *S. salamandra* would feature higher mechanical advantage (output bite force/input jaw muscle force), reflecting adaptation towards efficiently converting muscle to bite force, whereas the skull of *N. maculosus* would feature lower mechanical advantage, reflecting adaptation towards fast jaw movements used in suction feeding. *N. maculosus* exhibited both absolutely and relatively larger jaw muscles than *S. salamandra*. Elongation of the posterior (postorbital) region of the skull in *N. maculosus* as well as lateral expansion of the parietal and frontal provided increased attachment area and space for the jaw closing muscles. Furthermore, muscle pennation in *N. maculosus* (absent in *S. salamandra*) led to even greater discrepancy in muscle force between the two species. Although there were differences in the relative contributions of individual muscles between the taxa, differences in the proportions of the jaw opening versus closing muscles between the two taxa were minimal, rejecting H1.

Due to its relatively larger jaw closing muscles and greater degree of pennation, lever arm mechanics estimate that *N. maculosus* produced higher bite forces than *S. salamandra*, even when the latter is scaled to the same skull width. Mechanical advantage was slightly higher in *S. salamandra* during anterior bites, indicating improved efficiency for biting at the front of the jaws and possibly reflecting an adaptation for jaw prehension. However, mechanical advantage was higher during middle and posterior bites in *N. maculosus*. Thus, H2 was only partially supported. Relative to skull length, the moment arms of the jaw‐closing muscles in *S. salamandra* were comparable to (m. levator mandibulae longus) or longer than (m. levator mandibulae externus and internus) those of *N. maculosus*. It is possible that the much larger jaw closing muscles of *N. maculosus* may compensate for these relatively shorter moment arms. In contrast, the moment arms of the m. depressor mandibulae muscles were relatively longer in *N. maculosus* than in *S. salamandra*, due to its strongly anteriorly displaced jaw joint, thus allowing for more efficient jaw opening.

Our results suggest that skull geometry and jaw musculature of the terrestrial *S. salamandra* are not better adapted to produce stronger bites than the aquatic *N. maculosus*. Instead, other factors may be influencing muscle architecture and bite force. The two species have very different diets: terrestrial adults of *S. salamandra* eat relatively soft insects, larvae, slugs, spiders and earthworms (Bas Lopez et al. 1979; Balogová et al. 2015) whereas *N. maculosus* feeds on crayfish, other crustaceans, small fish, and insect larvae (Hamilton 1932). Larger jaw closing muscles and resulting high bite forces allow *N. maculosus* to crush harder crustacean shells. Furthermore, the physical properties of habitat may be playing a role in driving morphology: it has been proposed that *N. maculosus* uses a suction‐and‐biting hybrid feeding method, similar to that of cryptobranchids (Elwood and Cundall 1994; Heiss, Aerts, et al. 2013; Heiss, Natchev, et al. 2013; Fortuny et al. 2015; Matsumoto et al. 2024). *N. maculosus'*s larger jaw muscles may be an adaptation to overcome water resistance when opening and closing the jaws to capture prey. The pairing of low muscle mechanical advantage and large muscle size is also seen in crocodilians (Erickson et al. 2012; Gignac and Erickson 2016; Sellers et al. 2017; Heiss et al. 2023) and may be a common feature of aquatic feeding taxa that must capture elusive prey in water but also generate high bite forces. In contrast, adult *S. salamandra* do not need to overcome water resistance during feeding and feed on relatively soft prey, and therefore do not require enlarged jaw muscles. The lower bite forces in *S. salamandra* and its comparatively small teeth may be broadly comparable to frogs, which do not require chewing during their feeding (Pulah et al. 2021), though this comparison would need to be tested quantitively; apart from using their tongues to capture prey and one reference to prey‐crushing behaviour involving the tongue (Reilly 1996), little is known about food processing in *Salamandra*. Our data suggests that bite force in aquatic versus terrestrial taxa is not solely determined by feeding mechanism (suction vs. biting) but instead seems to reflect multiple demands on the feeding system (diet, overcoming water resistance, food processing behaviours at other stages in the feeding cycle, etc.).

### 4.2 Increased Skull Strength Is Correlated With Higher Bite Forces but Not Strictly With Habitat

We predicted that the skull of *S. salamandra* would experience lower peak stresses during biting than that of *N. maculosus* (H3), reflecting increased skull strength to resist the biting loads associated with terrestrial feeding. Although the life‐size model of *S. salamandra* did exhibit substantially lower peak stresses than *N. maculosus*, this was due to its much smaller jaw‐closing muscles. When scaled to the same surface area: muscle force ratio, the upper and lower jaws of *S. salamandra* exhibited higher peak stresses than those of *N. maculosus* under all feeding loads modelled, suggesting that when the effects of size are removed, the skull of *N. maculosus* is stronger than that of *S. salamandra*, rejecting H3. Thus, skull shape in the aquatic *N. maculosus* seems better adapted (in terms of increased strength) to resist these higher muscle and bite forces than that of the terrestrial *S. salamandra*. Alternatively, the aquatic habitat of *N. maculosus* may pose demands on skull shape other than resisting feeding‐induced loads, such hydrodynamic streamlining, sensory systems, and/or the need to produce suction, leading to increased safety factors in its skull.

Several trends in peak stress magnitudes were noted in both taxa. The lower jaw experienced higher peak stress at all bite points in *S. salamandra* and at five of six bite points in *N. maculosus*, suggesting greater strength of the upper jaw than the lower jaw under feeding loads. This was unsurprising, given that the upper jaw houses the brain and delicate sense organs, and its morphology is presumably under additional functional demands. Only during posterior unilateral biting did peak stress in the upper jaw exceed that in the mandible of *N. maculosus*. The upper jaws of both taxa seemed strongest (i.e., exhibited lowest peak stresses) during middle bites and weakest (i.e., exhibited highest peak stresses) during posterior bites. The lower jaw of *S. salamandra* experienced lowest peak stresses during uni‐ and bilateral posterior bites, suggesting the mandible of this taxon is strongest during posterior biting, possibly associated with food processing. In contrast, while the lower jaw of *N. maculosus* was also strongest during posterior bilateral biting, it experienced the highest peak stresses during posterior unilateral biting. Altogether, these results—as well as pronounced deformation of the upper and lower jaws and high stress in the palatopterygoid—suggest that the skull of *N. maculosus* is least well adapted for posterior unilateral bites compared to other bite load scenarios.

### 4.3 Terrestriality May Permit Increased Adaptation of Skull Shape for Feeding

Skulls—particularly upper jaws—are subject to multiple (possibly competing) functional demands, including feeding, housing and protecting the brain and sense organs, communication, combat/defense, and others. This is thought to result in functional trade‐offs, in which selection cannot simultaneously optimise performance (and by inference, morphology) for particular functions (Garland et al. 2022; Sansalone et al. 2024). Transitions between radically different habitats often demand changes in skull function and shape, imposing new constraints on morphology or, alternatively, releasing the skull from previous constraints. For example, amniotes generally feature narrower, taller skulls and smaller heads relative to body length than anamniotes (Janis and Keller 2001; Yu et al. 2026); this has been tied to a shift from buccal pumping to costal ventilation between anamniotes and amniotes, which is in turn linked to differences in postural support and changes in the shape of the axial column with increased terrestriality (Janis and Keller 2001; Yu et al. 2026). With the skull no longer constrained to a wide, flat shape needed for buccal pumping, this allowed for changes in skull morphology, jaw muscle arrangement and jaw mechanics that permitted dietary expansion in amniotes (Olson 1961; Carroll 1969), however, both *S. salamandra* (Simons et al. 2000) and *N. maculosus* (Brainerd et al. 1993) use buccal pumping, meaning that they do not reflect this particular change when used as analogues of the water–land transition.

Experimental data has demonstrated that reptiles feature higher strains in the bones of the skull roof overlying the brain (Porro et al. 2014; Ross et al. 2018) than mammals, in which the calvarial bones experience lower strains than the facial skeleton (Herring and Teng 2000; Thomason et al. 2001). It has been suggested that this is due to the need for the calvarial bones to protect the enlarged brains of mammals against infrequent impact loads that exceed loads generated by feeding. Thus, higher bone strains distributed more evenly throughout the reptilian skull may suggest that skull shape may be more optimised for maximum strength with minimum material during feeding than in mammals (Ross et al. 2018; Dutel et al. 2021; Sharp et al. 2023).

We proposed that the skull of *S. salamandra* would exhibit more evenly distributed stress under bite loads than that of *N. maculosus*, reflecting greater adaptation of skull shape to biting (H4). This is because we assumed that the aquatic habitat of *N. maculosus* imposes additional demands and constraints on its skull shape, such as hydrodynamic streamlining and generating suction, compared to the terrestrial *S. salamandra*. Qualitative comparisons of contour plots of the two taxa under similar feeding loads appeared to support H4: in general, more areas of the *S. salamandra* skull exhibited elevated stress compared to *N. maculosus*, in which the posterior skull roof and palate, and sections of the mandible featured very low stress. Thus, it would indeed seem that skull shape in *S. salamandra* is more optimised than *N. maculosus* to dissipate stress from bite loads, possibly due to release from the constraints associated with an aquatic habitat.

### 4.4 The Impact of Sutures on Skull Mechanics Is Complex

Sutures—fibrous joints between skull bones—are sites of bone growth during ontogeny. Experimental studies have demonstrated that sutures may perform a functional role by absorbing or modifying strain during feeding and other behaviours (Herring and Mucci 1991; Jaslow and Biewener 1995; Rafferty and Herring 1999; Herring and Teng 2000; Thomason et al. 2001). An advantage of FEA is that skulls can be modelled with and without sutures to isolate their mechanical impact; however, data from these sensitivity and validation studies suggest the functional role of sutures is complex and may vary between taxa. In some cases, inclusion of sutures in FEMs only impacted local strain patterns and magnitudes, particularly close to sutures (Wang et al. 2010; Sharp et al. 2023), whereas in other taxa including sutures resulted in changes to global stress and strain patterns in the cranium (Moazen et al. 2009; Bright 2012; Curtis et al. 2013; Jones et al. 2017) and mandible (Porro et al. 2011; Reed et al. 2011). Nearly all studies report that inclusion of sutures results in increased strain magnitudes. Because sutures are time‐consuming and labour‐intensive to incorporate into FEMs, it is crucial to understand their effect on the mechanical behaviour of the skull.

Peak stress magnitudes were higher in models of *N. maculosus* and *S. salamandra* that included sutures compared to solid models, rejecting H5 and—because stress increases proportionally with increased strain—in line with results from FEA studies on other taxa. Stress patterns in the mandible were nearly identical between models with and without sutures for both taxa. As both species contained only four bones in the lower jaw, with the dentary forming the majority of the mandible, it is possible that mandibular sutures have a less pronounced impact on their mechanical behaviour compared to the lower jaws of reptiles, which have been shown to be sensitive to the inclusion of sutures (Porro et al. 2011; Reed et al. 2011). However, in terms of other performance metrics, there were differences between the two taxa. Bite force increased in both species when the skull was modelled without sutures, which is expected given that the entire structure is stiffer and better able to transmit force. However, bite forces increased substantially more in the solid *S. salamandra* model than in the solid *N. maculosus* model compared to their counterparts with sutures. Furthermore, the upper jaw of *S. salamandra* featured more pronounced changes in global stress patterns compared to the suture model than that of *N. maculosus*. Our results and those of previous studies demonstrated that the way sutures impact skull mechanical performance under feeding loads varies between taxa and may be linked to differences in shape (including the size of the brain and eyes), function, ecology and/or ontogeny. Thus, caution must be taken in terms of the questions that can be addressed using FE models that do not include sutures, and studies on more salamander taxa with and without sutures are needed.

### 4.5 Comparisons of Skull Mechanics in *Necturus* and *Salamandra* to Other Taxa

There have been relatively few FEA studies of salamander skulls compared to reptiles and mammals, including models of the upper jaws (no mandibles) of the aquatic Chinese giant salamander, *Andrias davidianus* (Fortuny et al. 2015), the terrestrial Siberian salamander, *Salamandrella keyserlingii* (Zhou et al. 2017), and the California giant salamander, *Dicamptodon ensatus* (Fortuny et al. 2016; Supporting data of Zhou et al. 2017). The latter species features both terrestrial, metamorphosed adults and aquatic adults that retain gills, but it is unspecified to which type the individual in the study pertains. *A. davidianus* was modelled under bilateral and unilateral bites at anterior and posterior positions. *S. keyserlingii* was modelled under bilateral bites (anterior and posterior). *D. ensatus* was modelled under anterior (unilateral and bilateral) and posterior (bilateral) bites.

As with both of our study taxa, *A. davidianus* featured highest stresses in the posterior parietals, squamosals and quadrates under all bite loads. *A. davidianus* exhibited substantially higher stresses during unilateral and posterior biting, with posterior unilateral bites resulting in the highest stresses (Fortuny et al. 2015), similar to *N. maculosus* and *S. salamandra*. During both anterior and posterior bilateral bites, stress patterns in the skull of *A. davidianus* more closely resembled those in *S. salamandra*—with regions of high stress around the nasal‐frontal and vomer‐parasphenoid that shifted posteriorly with bite point—than those in *N. maculosus*. During posterior unilateral bites, *A. davidianus* exhibited high stress in the working side preorbital region, squamosal, quadrate, vomer‐parasphenoid contact and posterior ramus of the pterygoid. This strongly resembled stress patterns we found during posterior unilateral biting in *S. salamandra* and was very different from the pattern in *N. maculosus*, in which the highest stresses occur in the working side palatopterygoid*. A. davidianus* and *S. salamandra* both lack a bony connection between the tooth‐bearing maxilla and the pterygoid and quadrate, and loads between the muscle attachment sites and posterior bite points must travel through the skull roof and central part of the palate. However, the tooth‐bearing palatopterygoid of *N. maculosus* strongly joins the quadrate, allowing loads (resulting in high stress) to travel through this structure.

Peak stresses in the upper jaw of *S. keyserlingii* were slightly higher than values recorded in *N. maculosus* and, as in both of our study taxa, *S. keyserlingii* exhibited higher stresses during posterior biting compared to anterior biting (Zhou et al. 2017). Also, as in *N. maculosus* and *S. salamandra*, *S. keyserlingii* exhibited elevated stresses in the posterior parietals, squamosals and quadrates at both bite points tested. Interestingly, stress patterns in the middle and posterior portions of the skull are very similar during anterior and posterior biting in *S. keyserlingii*, with a pronounced “stress belt” around the frontal‐parietal suture that continues onto the palate at the parasphenoid‐orbitosphenoid suture; the only difference between stress distributions between the two loading scenarios in *S. keyserlingii* were elevated stresses in the premaxilla during anterior bites. In contrast, both of the taxa in our study (as well as *A. davidianus* in Fortuny et al. 2015) showed markedly different stress patterns between anterior and posterior bilateral biting.

As with all other taxa discussed, FE models of *D. ensatus* (Fortuny et al. 2016; Supporting data of Zhou et al. 2017) featured elevated stresses in the posterior skull roof, palate, squamosals and quadrate under all bite loads. As with *S. keyserlingii, D. ensatus* did not feature pronounced differences in stress patterns between anterior and posterior bilateral biting, with a pronounced stress belt around the frontal‐parietal suture (Zhou et al. 2017). In contrast, during anterior unilateral bites, stress patterns in *D. ensatus* change markedly, with a band of high stress in the posterior skull roof and patterns in the anterior snout and palate resembling those exhibited by *S. salamandra*.

While FEMs of the upper jaws of all five taxa show some similarities—in particular elevated stresses in the posterior skull roof, squamosals and quadrates due to muscle and joint reaction forces under feeding loads—there are also notable differences. The skull of *S. keyserlingii* seems to respond similarly despite changes in bite position, unlike the other taxa. It should be noted that this taxon is unique among species tested in featuring an anteroposteriorly elongate median fontanelle along the skull roof. Additionally, stress distributions in the skulls of both *D. ensatus* and *A. davidianus* more closely resemble those in *S. salamandra* than *N. maculosus*. As noted earlier, it is unclear whether the *D. ensatus* skull analysed by Fortuny et al. (2016) is from a metamorphosed or aquatic adult; however, the similar mechanical performance between the skulls of the large, aquatic *A. davidianus* and the small, terrestrial *S. salamandra* is unexpected. This could be due to greater structural similarities between the skulls of these two taxa, that is, loss of direct contact between the tooth‐bearing bones and the jaw joint, compared to *N. maculosus*. Alternatively, differences in feeding mechanism between *A. davidianus* and *N. maculosus* could explain differences in mechanical response of the skull. Cryptobranchids possess a poorly ossified hyobranchial apparatus and rely more on movements of the mandible during suction feeding (Matsumoto et al. 2024). In contrast, *Necturus* possesses a heavily ossified hyobranchial apparatus that it uses to generate suction (Deban and Wake 2000). Despite a shared habitat, differences between these two aquatic taxa in skull structure, diet and feeding mechanism may result in substantial variations in mechanical behaviour of the skull. With only five species of salamander studied using FEA, and major differences in body size, habitat and/or diet between them, further study of other salamanders is essential.

To our knowledge, no FEMs of salamander lower jaws have been published outside the present study, but we can compare our results from the lower jaws of *S. salamandra* and *N. maculosus* to those of non‐salamander taxa. During unilateral biting, both salamander taxa exhibited greater deformation of the balancing side manidibular ramus—upward and medial bending, and inversion (medial twisting) of the dorsal margin—compared to the working side, which primarily experienced dorsoventral bending. Additionally, in both taxa, the mandibular symphysis was deflected towards the working side during unilateral bites. Similar deformation of the mandible during unilateral biting occurs in *Alligator* (Porro et al. 2011, 2013), in which in vivo experiments and FEA reveal that the balancing side undergoes strong dorsal and medial bending, and inversion of the tooth row, while the working side undergoes dorsoventral and medial bending; however, the salamander models did not exhibit the pronounced eversion (outward twisting) of the working side tooth row seen in *Alligator*. Anthropoid primate mandibles exhibit dorsal bending and lateral bending and/or inversion of the tooth row on the balancing side, and dorsal bending, and medial bending and/or eversion of the tooth row on the working side (Panagiotopoulou et al. 2020; Smith et al. 2021). Thus, while all three groups—salamanders, crocodilians, and anthropoid primates—share some similarities in mandibular deformation during biting, there are important differences attributable to anatomical variation. Crocodilians and salamanders both possess relatively longer mandibles than primates, resulting in greater importance of bending than torsion during biting in these groups. Additionally, the masseter muscles of mammals attach onto the lateral side of the mandible, resulting in eversion of the angle of the mandible (Hylander 1979). In contrast, the enlarged pterygoideus muscles of crocodilians attach on the ventromedial aspect of the mandible, with pterygoideus dorsalis wrapping around the ventral margin of the mandible to attach to the ventrolateral aspect (Holliday et al. 2013), inducing inversion of the ventral margin of the mandible during contraction. In the two salamander taxa examined in this study, the jaw‐closing muscles inserted onto the dorsal aspect of the posterior mandible, with no attachments on its ventromedial or ventrolateral aspects. For this reason, torsion in the salamander mandible may not be as pronounced as in mammals or crocodilians.

### 4.6 Study Limitations

Some limitations to the present study should be noted. Although studying living relatives provides important insights into aspects of the tetrapod water–land transition, the extant phylogenetic bracket encompassing stem tetrapods—including living sarcopterygian fishes, such as coelacanths and lungfish, and extant amphibians—is very broad, and both bracketing clades are morphologically derived compared to ancestral forms. Furthermore, stem tetrapods preserve unique morphologies not represented in modern relatives. Therefore, although in many ways salamanders provide important insights into the adaptations required for the water–land transition (Heiss et al. 2018; Schwarz et al. 2023) they are not perfect functional analogues for early tetrapods. Furthermore, our sample consists of only two salamanders due to the availability of specimens for scanning and dissection, and time available for the project; this limits the broad applicability of our observations across salamanders, but provides groundwork for upcoming future studies on a wider sample.

Our verbal descriptors of deformation and stress distribution are approximations and, as noted elsewhere (Chalk et al. 2010; Porro et al. 2011; Ross et al. 2011), the FEMs with their specific geometries, material properties, boundary conditions and their resulting deformation and stress regimes are our hypotheses of the mechanical behaviour of the *S. salamandra* and *N. maculosus* skulls during biting. In this study, we only modelled a single gape angle and six bite loads, whereas living salamanders can load their skulls in many different ways. We assumed that all the jaw‐closing muscles contract simultaneously and maximally, and we did not account for muscle length‐tension curves at the modelled gape. Because bone and suture material properties are not available for salamander skull bone, we used available data for *Alligator*, and our properties are not taxon specific. We did not model teeth separately and used homogeneous, isotropic properties for bone and sutures, although most FEA studies that model sutures or other soft tissues also make this assumption. Nonetheless, these assumptions could impact model behaviour as demonstrated by Porro et al. (2011). Lastly, due to the smaller size of *S. salamandra* compared to *N, maculosus*, it is possible that sone of the smallest posteriormost teeth of *S. salamandra* were not resolved in the scan and the position of the posterior bite point in this taxon may be further anterior than it could be in reality.

To our knowledge, in vivo bite forces are not available for *S. salamandra*. Our bite force estimates (Tables 3 and 4) for our *N. maculosus* specimen with a head length of 34.3 mm were 7.72 N (lever arm mechanics) and 9.97 N (FEA) during a posterior bite. This is less than in vivo bite force measured in a *N. maculosus* specimen with a head length of 34.4 mm of 19.9 N (Anthony Herrel pers. comm. 2026). Because specimens were stained and scanned prior to current staining procedures that incorporate buffers to prevent soft‐tissue shrinkage (see following paragraph), the most likely explanation is muscle shrinkage during staining. Therefore, values for maximal bite forces and peak stresses reported here should be seen as conservative estimates of these values for *N. maculosus* and *S. salamandra* individuals of this size.

Due to the lack of available material properties for amphibian cartilage, the Meckel's cartilage of *N. maculosus*—which forms the lower articulation surface of the jaw joint and some of the adductor muscles attach to—could not be modelled with accurate material properties. Obtaining such material properties could open new avenues of study and improve the accuracy of FEMs, especially for taxa with a higher proportion of cartilage in their skulls.

This work was carried out in 2017–2018 and predates more recently published guidance on incorporating buffers into Lugol's solution to stabilise pH and reduce soft‐tissue shrinkage (Hedrick et al. 2018; Dawood et al. 2021; Gray et al. 2023); we used higher concentrations of iodine than is now recommended practice. It is possible shrinkage of soft tissues occurred, so we report both raw muscle mass as well as relative muscle mass measurements, but these should not be interpreted as in vivo values. Iodine cannot stain connective tissues, so tendinous structures cannot be visualized with diceCT; this was partially mitigated by recording muscle attachment sites and taking measurements during physical dissections.

## 5 Conclusions

This study demonstrated major differences in skull anatomy, including skeletal and soft tissues, in two salamander taxa: the terrestrial salamandrid *Salamandra salamandra* and the aquatic proteid *Necturus maculosus*. We found that, even when accounting for differences in head size, *N. maculosus* possessed substantially larger muscles and exhibited greater mechanical advantage at most bite points than *S. salamandra*, contrary to our predictions. Although the life‐size FEM of the *S. salamandra* skull featured lower peak stresses than that of *N. maculosus*, when scaled to the same surface area: muscle force ratio, the *S. salamandra* model exhibited higher peak stresses than the *N. maculosus* model, again contrary to our prediction. Our results did support our prediction that skull shape in *S. salamandra* would more strongly reflect adaptation to resist biting loads than in *N. maculosus*, potentially due to additional functional demands imposed by the aquatic habitat of the latter taxon. Lastly, the inclusion of sutures resulted in increased peak stress magnitudes compared to solid models in both species, although the impact of sutures on bite force and stress distributions differed between the two taxa, highlighting the complex and varied functional role of skull sutures.

From a methodological perspective, our study highlights how combining physical and digital dissection allows new insights into studies of muscle architecture: for instance, the m. depressor mandibulae and m. depressor mandibulae posterior were fused in physical dissections of both taxa, but could be clearly discerned in diceCT data, illustrating the utility of integrating these techniques.

It is noteworthy that our results rejected many of our hypotheses, which were based on the idea that since *S. salamandra* is primarily terrestrial, it cannot use suction feeding to capture and ingest prey (as *N. maculosus* does) and must rely on biting alongside tongue prehension. Our expectation was that skull skeletal and muscle anatomy in *S. salamandra* would result in higher bite forces and increased skull strength under bite loads. Our results demonstrated that terrestrial habitat does not necessarily mandate increased bite forces and skull strength. As already noted, there are major differences between the two species in diet, with *N. maculosus* consuming hard invertebrate prey. The two taxa also achieve different maximum body sizes: *N. maculosus* can attain a maximum body length of over 40 cm (Gans and Nussbaum 1981) whereas *S. salamandra* can reach lengths of over 30 cm (Griffiths 1996). Additionally, comparisons with previously published FEMs of the aquatic *A. davidianus* (Fortuny et al. 2015) revealed greater similarity in terms of stress patterns with the terrestrial *S. salamandra* than the aquatic *N. maculosus*. This could be due to closer structural similarity in the skulls of *A. davidianus* and *S. salamandra*, dietary differences between *A. davidianus* and *N. maculosus*, and/or differences in how *A. davidianus* and *N. maculosus* generate suction. Although our study provides novel and much‐needed insights into salamander skull function, it is difficult to pinpoint which factors—body size, habitat, jaw mechanism, diet, or skull structure (perhaps constrained by development or phylogeny)—are the primary drivers of skull shape in salamanders. This highlights the requirement of a larger sample size encompassing greater taxonomic, morphological and dietary diversity of living salamanders to better understand the link between skull shape and performance, as well as emphasising the need for caution when making inferences about changes in form and function across the tetrapod water–land transition based on only one or two extant relatives.

## Author Contributions

**Julia R.S. May:** investigation, methodology, visualisation, writing – original draft preparation. **Laura B. Porro:** conceptualisation, data curation, funding acquisition, investigation, methodology, resources, supervision, visualisation, writing – original draft preparation. **Emily J. Rayfield:** conceptualisation, funding acquisition, supervision, writing – review and editing.

## Conflicts of Interest

The authors declare no conflicts of interest.

## Supporting information


Supporting File

## Data Availability

The CT scans used in this project are available on MorphoSource. The FE models are available through the corresponding author and can be accessed in the free Strand7 Viewer.
