# Supplementary material for: Comparative Morphology and Mechanics of Aquatic and Terrestrial Feeding Salamanders
Source: J Morphol. 2026 Sep 20;287(9):e70172. doi: 10.1002/jmor.70172 (PMC13589827; doi:10.1002/jmor.70172)
Supplement: Supplementary file 1 [file JMOR-287-e70172-s001.docx]

# Supplementary Tables

The following three supplementary data tables contain all of the metrics from both the physical and digital dissections of *S. salamandra* and *N. maculosus* conducted in this study. They are divided into separate tables for ease of reading. All values are to two significant figures except for muscle masses, because of the small size of the muscles in *S. salamandra*.

**Supplementary Table 1:** Muscle volumes and lengths from digital dissection.

| Taxon | Muscle | Volume (mm^3^) | Muscle Length (mm) |
| --- | --- | --- | --- |
| *N. maculosus* | Levator internus Left | 515.08 | 26.67 |
|  | Levator internus Right | 492.36 | 25.15 |
|  | Levator externus Left | 677.96 | 23.5 |
|  | Levator externus Right | 687.08 | 22.73 |
|  | Levator longus Left | 262.20 | 24.51 |
|  | Levator longus Right | 244.45 | 23.8 |
|  | Depressor Left | 89.72 | 17.55 |
|  | Depressor Right | 92.10 | 16.58 |
|  | Dep. posterior Left | 274.68 | 22.5 |
|  | Dep. posterior Right | 278.45 | 21.92 |
| *S. salamandra* | Levator internus Left | 7.78 | 6.22 |
|  | Levator internus Right | 7.07 | 6.22 |
|  | Levator externus Left | 9.04 | 5.95 |
|  | Levator externus Right | 8.94 | 5.95 |
|  | Levator longus Left | 12.36 | 11.6 |
|  | Levator longus Right | 10.67 | 11.6 |
|  | Depressor Left | 2.74 | 6.68 |
|  | Depressor Right | 2.60 | 6.68 |
|  | Depressor posterior Left | 3.91 | 6.68 |
|  | Depressor posterior Right | 3.22 | 6.68 |

**Supplementary Table 2:** *N. maculosus* masses and measurements from physical dissection.

| Muscle | Characteristic | Description/Measurement |
| --- | --- | --- |
| Levator internus | Shape | Fan-shaped |
|  | Insertion type | Tendinous |
|  | Pennation type | Unipennate |
|  | Pennation angle (°) | 27.06 |
|  | Total muscle length (mm) | 25.57 |
|  | Averaged fibre lengths before removal (mm) | 10.185 |
|  | Measured fibre length post-dissection (mm) | 8.64 |
|  | Tendon length (mm) | 1.63 |
|  | Mass (g) | 0.4621 |
| Levator externus | Shape | Fan-shaped |
|  | Insertion type | Aponeurotic |
|  | Pennation type | None |
|  | Total muscle length (mm) | 20.16 |
|  | Averaged fibre lengths before removal (mm) | 14.53 |
|  | Averaged fibre lengths post-dissection (mm) | 13.76 |
|  | Aponeurosis length (mm) | 3.68 |
|  | Mass (g) | 0.6255 |
| Levator longus | Shape | Fusiform |
|  | Insertion type | Aponeurotic |
|  | Pennation type | none |
|  | Total muscle length (mm) | 16.06 |
|  | Fibre length before removal (mm) | 16.06 |
|  | Fibre length after dissection (mm) | 17.69 |
|  | Aponeurosis length (mm) | 1.66 |
|  | Mass (g) | 0.1812 |
| Depressor (fused with Depressor posterior) | Shape | parallel-fibred fan |
|  | Insertion type | Tendinous |
|  | Pennation type | none |
|  | Total muscle length (mm) | 11.41 |
|  | Fibre length before removal (mm) | not taken |
|  | Fibre length after dissection (mm) | 14.53 |
|  | Tendon length (shared w. D. post) (mm) | 4.04 |
|  | Mass (g) | est. 0.0538 |
| Depressor posterior (fused with Depressor) | Shape | Fan-shaped |
|  | Insertion type | Tendinous |
|  | Pennation type | Unipennate |
|  | Pennation angle (°) | 14.86 |
|  | Total muscle length (mm) | 15.15 |
|  | Fibre length before removal (mm) | not taken |
|  | Fibre length after dissection (mm) | 7.68 |
|  | Tendon length (shared w. Dep.) (mm) | 4.04 |
|  | Mass (g) | est. 0.0538 |
| Specimen measurements | Head mass (g) | 28.42 |
|  | Tip-of-snout-to-gills length (mm) | 43.54 |
|  | Pre-orbital length (mm) | 12.71 |
|  | Mouth tip-to-corner length (mm) | 12.99 |
|  | Tip-of-snout width (mm) | 17.74 |
|  | Just-before-gills width (mm) | 35.91 |
|  | Over-the-orbit head depth (mm) | 14.92 |
|  | Just-before-gills head depth (mm) | 23.56 |

**Supplementary Table 3:** *S. salamandra* masses and measurements from physical dissection.

| Muscle | Characteristic | Description/Measurement |
| --- | --- | --- |
| Levator internus | Shape | Strap |
|  | Insertion type | Aponeurotic |
|  | Pennation type | none |
|  | Total muscle length (mm) | 6.22 |
|  | Fibre length after dissection (mm) | 4.92 |
|  | Aponeurosis length (mm) | 1.30 |
|  | Mass (g) | 0.0054 |
| Levator externus | Shape | Fan-shaped |
|  | Insertion type | Aponeurotic |
|  | Pennation type | None |
|  | Fibre length after dissection (mm) | 5.95 |
|  | Aponeurosis length (mm) | Too small to measure accurately |
|  | Mass (g) | 0.0092 |
| Levator longus | Shape | Strap |
|  | Insertion type | Tendinous |
|  | Pennation type | none |
|  | Total muscle length (mm) | 11.60 |
|  | Fibre length after dissection (mm) | 9.77 |
|  | Tendon length (mm) | 1.83 |
|  | Mass (g) | 0.0076 |
| Depressor (fused with Depressor posterior) | Shape | Fan-shaped |
|  | Insertion type | Aponeurotic |
|  | Pennation type | None |
|  | Total muscle length (mm) | 7.13 |
|  | Fibre length after dissection (mm) | 5.72 |
|  | Aponeurosis length (shared w. D. post) (mm) | 0.89 |
|  | Mass (g) | est. 0.0066 |
| Depressor posterior (fused with Depressor) | Shape | Fan-shaped |
|  | Insertion type | Aponeurotic |
|  | Pennation type | none |
|  | Total muscle length (mm) | 7.13 |
|  | Fibre length after dissection (mm) | 6.68 |
|  | Aponeurosis length (shared w. Dep.) (mm) | 0.89 |
|  | Mass (g) | est. 0.0066 |
| Specimen measurements | Head mass (g) | 2.96 |
|  | Tip-of-snout-to-axial-vertebra length (mm) | 20.20 |
|  | Pre-orbital length (mm) | 7.62 |
|  | Mouth tip-to-corner length (mm) | 12.81 |
|  | Tip-of-snout width (mm) | 7.90 |
|  | Across-jaw-joint width (mm) | 18.72 |
|  | Over-the-orbit depth (mm) | 10.15 |
|  | Maximum head depth (mm) | 11.10 |
